# Generation and Validation of Monoclonal Antibodies Suitable for Detecting and Monitoring Parvovirus Infections

**DOI:** 10.3390/pathogens11020208

**Published:** 2022-02-04

**Authors:** Claudia Tessmer, Claudia Plotzky, Jana Fees, Hendrik Welsch, Rebecca Eudenbach, Martin Faber, Alicia Simón, Assia Angelova, Jean Rommelaere, Ilse Hofmann, Jürg P. F. Nüesch

**Affiliations:** 1 Genomics and Proteomics Core Facility, Unit Antibodies, German Cancer Research Center (DKFZ), 69120 Heidelberg, Germany; c.tessmer@dkfz.de (C.T.); i.hofmann@dkfz.de (I.H.); 2 Program Infection, Inflammation and Cancer, Division Virus-Associated Carcinogenesis (F170), German Cancer Research Center (DKFZ), 69120 Heidelberg, Germany; c.plotzky@dkfz.de (C.P.); jf1403@gmx.de (J.F.); hwe21@web.de (H.W.); Rebecca.eudenbach@gmx.de (R.E.); m.faber@klinikum-braunschweig.de (M.F.); alisimon@ucm.es (A.S.); 3 Program Infection, Inflammation and Cancer, Clinical Cooperation Unit Virotherapy (F230), German Cancer Research Center (DKFZ), 69120 Heidelberg, Germany; a.angelova@dkfz-heidelberg.de (A.A.); j.rommelaere@dkfz.de (J.R.)

**Keywords:** parvoviruses, monoclonal antibodies, NS1, diagnostic tools, generation of monoclonal antibodies for recognizing parvoviral NS1 proteins

## Abstract

For many applications it is necessary to detect target proteins in living cells. This is particularly the case when monitoring viral infections, in which the presence (or absence) of distinct target polypeptides potentially provides vital information about the pathology caused by the agent. To obtain suitable tools with which to monitor parvoviral infections, we thus generated monoclonal antibodies (mAbs) in order to detect the major non-structural protein NS1 in the intracellular environment and tested them for sensitivity and specificity, as well as for cross-reactivity towards related species. Using different immunogens and screening approaches based on indirect immunofluorescence, we describe here a panel of mAbs suitable for monitoring active infections with various parvovirus species by targeting the major non-structural protein NS1. In addition to mAbs detecting the NS1 of parvovirus H-1 (H-1PV) (belonging to the *Rodent protoparvovirus 1* species, which is currently under validation as an anti-cancer agent), we generated tools with which to monitor infections by human cutavirus (CuV) and B19 virus (B19V) (belonging to the *Primate protoparvovirus 3* and the *Primate erythroparvovirus 1* species, respectively, which were both found to persistently infect human tissues). As well as mAbs able to detect NS1 from a broad range of parvoviruses, we obtained entities specific for either (distinct) members of the *Rodent protoparvovirus 1* species, human CuV, or human B19V.

## 1. Introduction

Autonomous, vertebrate parvoviruses (PVs) are icosahedral, non-enveloped particles of approximately 24 nm in diameter, with a 5 kb linear single-stranded DNA as their genome. This small nucleic acid encodes the VP proteins composing the capsid shell and a small number of nonstructural (NS) proteins, necessary to reprogram the host cell in order to support viral DNA amplification, packaging, and spreading. In an exposed organism, tissue tropism can be restricted through a limiting viral receptor at the cell surface, required for virus attachment and entry as well as the delivery into the nucleus and decapsidation of the single-stranded DNA [1]. Moreover, before viral regulatory proteins are produced to take control of the host cell, the single-stranded DNA genome has to be converted into a double-stranded transcription template in an S-phase-dependent process that limits productive infections to proliferating tissues [2]. Thus, it is possible that these viruses are capable of persisting in tissues/cells as innocuous passengers.

While rodent PVs, including H-1PV, were initially discovered to be opportunistic infectants of human-cancer-derived cell lines, and are currently validated as therapeutics for cancer treatments, other members of this virus family *(Parvoviridae)* have been recognized as (potential) pathogens that infect mammals (PPV, FPV/CPV) and humans (B19V, PARV4, HBoV1-4, CuV, and BuV), respectively [3,4,5,6,7]. Interestingly, besides causing acute diseases, including fetal abortions, some members have been found to persist in various tissues, potentially causing chronic diseases [8], as well as in cancer tissues [9,10], potentially as opportunistic infectants similar to the previously detected rodent protoparvoviruses [3]. Although the detection of viral DNA and transcripts in tissue and body fluids is achieved through very sensitive and specific methods, complementary approaches, such as the identification of specific proteins (functions) and consequently their impact on the affected cells and tissues, can be useful. This is possible using immunological assays, thereby identifying distinct viral proteins in cell compartments and/or co-localizations/interactions with cellular partner proteins. Such investigations might indeed lead to a better understanding of tissue tropism and their potential disease associations during persistent infections on the one hand, and might validate the impact of oncolytic PVs as anti-cancer agents on the other hand.

Besides the two capsid proteins VP1 and VP2, which together compose the capsid shell, parvoviruses are able to produce a number of non-structural proteins (NS), which are involved in multiple aspects of the viral life cycle, thereby ensuring progeny particle production and spreading. For rodent protoparvoviruses, there are four polypeptides produced from the early P4 promoter (NS1 and three forms of NS2) and one regulatory protein (SAT) generated from the P38 promoter [1,11]. Among these regulatory parvoviral proteins, the large non-structural NS1 protein is the most abundant and representative actor, and therefore serves best as a diagnostic marker of a productive infection. The NS1 protein is a multifunctional regulatory polypeptide involved in many processes, and is necessary for parvovirus propagation and spreading. As the key regulatory protein it becomes apparent at very early stages of infection, most likely to set up replication centers that, at later stages, will grow into large nuclear APAR bodies [12]. In the replicative phase NS1 drives DNA amplification, and *trans* activates the P38 promoter to ensure capsid protein production and packaging [1]. In addition, NS1 coordinates intracellular signaling through direct interaction with casein kinase 2 alpha [13] and the PKCη-accessory protein radixin [14], and facilitates viral egress through co-localization [15]. At late(r) stages of infection NS1 is responsible for selectively destroying and reorganizing cytoskeleton filaments, leading to remarkable cytopathic changes and culminating in cellular collapse and the disintegration of the plasma membrane, as indicated by the formation of lysis plaques [16]. To fulfill all these functions, NS1 is able to interact and interfere with many different host cell proteins and mechanisms, accounting for the cytotoxicity of the viral product [17]. In accordance with its slow turnover, which is in contrast to NS2 proteins (half-life > 6 h vs. 20 min), and accumulation through all stages of infection [18], which is in contrast to SAT [11], NS1 appears to be a perfect candidate with which to distinguish a potential productive infection from the silent accumulation of virions in a non-productive environment.

Similar to rodent protoparvoviruses, the newly identified primate protoparvoviruses, bufavirus (BuV), tusavirus (TuV), and cutavirus (CuV), encode, besides VPs, a large non-structural protein, NS1, and are recognized by a middle open reading frame, which could encode for an additional regulatory protein [7]. B19V was shown to produce two additional small regulatory proteins of 11 kDa and 7.5 kDa, respectively, together with the large 78 kDa NS1 polypeptide. Similar to rodent PV NS1, B19V NS1 contains a nuclear localization signal, has a DNA-binding/endonuclease domain and an NTP-binding motif in the central region, and has a putative trans-activation domain at the C-terminus. Thus, it is responsible for multiple different activities and is necessary for progeny particle production and spreading, including viral DNA replication and the induction of cell disturbances, such as cell cycle arrest, DNA damage responses, and the induction of apoptosis. It performs many different functions throughout the replication cycle, both at early and late stages of infection [7,19].

Indeed, monoclonal antibodies recognizing NS1 from B19V and the minute virus of mice (MVM), respectively, have been generated in the past and have been proven to be excellent tools not only to monitor the viral polypeptide in its cellular environment but also to capture and purify the polypeptide for functional analyses [20,21]. Therefore, to discriminate silent from productive parvoviral infections, we were particularly interested in generating such tools for various (potential) oncolytic PVs, including H-1PV, together with parvoviruses known to cause persistent infections in humans. This is the case of B19V [8] and the recently identified primate protoparvovirus CuV [9,10]. Besides acute illnesses, such as fifth disease and hydrops fetalis, B19V is associated with chronic diseases, including arthritis and arthralgias, where it can lead to painful swollen joints potentially initiated through the recurrent production of inflammatory cytokines [22]. CuV was originally found in stool samples, and was additionally detected in malignant melanoma and T-cell lymphoma [9,10], where it could cause opportunistic infections of cancer cells, as previously documented for a variety of rodent protoparvoviruses, including H-1PV [3].

Thus, we generated a number of mAbs using antigens for H-1PV, CuV, and B19V NS1, respectively, and characterized them for their specificity/cross-reactivity towards different parvovirus species and, consequently, their suitability to detect viral activity in human tissues through immunological assays.

## 2. Results

### 2.1. Antigen Design and Screening Approach to Generate mAbs Specific for NS1 from Rodent PVs, Human CuV, and b19v

**Figure 1:**
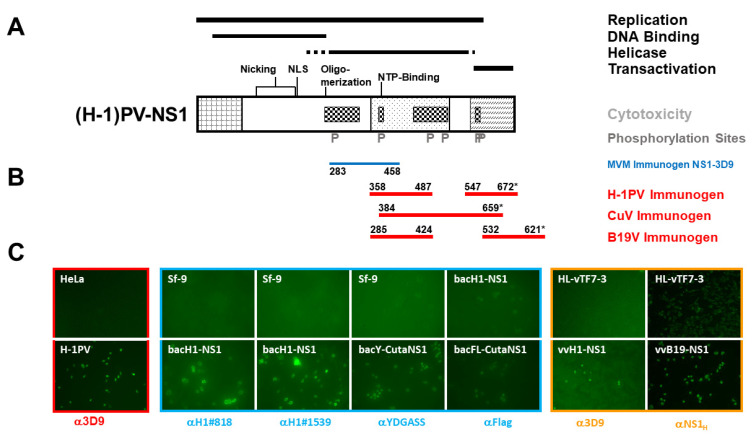
Immunogens and screening approaches. (**A**) Schematic representation of rodent parvovirus NS1 with the common N-terminus of NS1 and NS2 on the left (cross-hatched square), the conserved region with the SV40 T antigen in the middle (dotted square), and the transactivation domain on the right (hatched square). Functional domains, conserved motifs for ATP binding, nicking, oligomerization, and nuclear translocation, and identified phosphorylation sites are indicated [25]. (**B**) Location of the polypeptides used for immunizations, (*) denotes the termination codon of NS1. (**C**) Assessment of the suitability of the different screening approaches to identify mAbs suitable to detect the respective proteins by immunofluorescence. *Framed red:* to screen for mAbs recognizing rodent PV (H-1PV) NS1, HeLa cells were infected or not with H-1PV, fixed after 24 h with formaldehyde, and the detection of H1-NS1 was ascertained using previously established mAb 3D9 [18,26]. *Framed blue:* Sf-9 cells were infected (or not) with rBacH1-NS1, rBac-yCutaNS1, or rBacFL-CutaNS1, respectively. To determine the potential identification of mAbs detection was performed with mAb H1#818 for bacH1-NS1, or polyclonal antiserum αYDGASS (bacY-CutaNS1) and αFlag (bacFL-CutaNS1), respectively. *Framed brown:* HeLa cells were infected with vTF7-3 (a vaccinia virus producing the T7 polymerase) or vTF7-3+vvH1-NS1 or vvB19-NS1, respectively, and detection was performed with mAb 3D9 or polyclonal antiserum NS1_H_ [23].

To obtain the mAbs we considered distinct amino acid regions in NS1, which are exposed and therefore accessible to interact with cell proteins and/or other reagents, such as antibodies. In addition, our goal was to obtain mAbs with diverse specificity: (i) reagents able to detect NS1 from a broad spectrum of PVs and (ii) mAbs with a distinct species specificity, in order to selectively identify oncolytic PVs in the host without cross-reactivity through the detection of human (proto)parvoviruses and vice versa. Thus, we decided to choose a combination of (a) conserved domain(s) and (a) variable region(s) for immunization. For the prototype strain of the minute virus of mice (MVMp), the functional domain structure of NS1 together with target phosphorylation sites together with sequence alignments between different rodent PV species have been determined [17], and were used to identify suitable polypeptides for immunizations. Accordingly, we produced, for H-1PV, a polypeptide (aa 358–487) derived from the conserved helicase domain, containing ATP and Mg^2+^ binding sites, and a second polypeptide (aa 547–672) corresponding to the variable C-terminus comprising the *trans* activation domain (Figure 1A). It is noteworthy that, for MVMp, similar polypeptides were successfully used to produce polyclonal antisera αNS1_H_ and MK3/SP8, respectively, allowing the detection of NS1 by immunofluorescence and Western blotting [23,24]. To produce suitable peptides for the generation of CuV and B19V NS1 antibodies, we performed sequence alignments between H-1PV NS1 and CuV NS1 (Accession KT868814.1 AMS35105.1) and H-1PV NS1 and B19V NS1 (Accession NC_000883.2). As only limited sequence identity was detected between the helicase domains of B19V NS1 and H-1PV NS1 we additionally assessed potential similarities of the secondary structures between the two polypeptides (Figure S1). Accordingly, we produced a single polypeptide spanning aa 384–659 of CuV NS1 and two separate polypeptides corresponding to aa 285–424 and aa 523–621 of B19V NS1 (Figure 1B). The polypeptides were produced in bacteria, isolated from inclusion bodies, and purified by SDS-PAGE. Immune-competent mice were then challenged by multiple injections using species-specific polypeptides (one for CuV NS1 and two for H-1PV- and B19V-NS1, respectively), and after evidencing a significant immune response the mice were sacrificed; the lymphocytes were isolated and fused to SP2/0 myeloma cells to generate hybridomas potentially producing mAbs recognizing H-1PV, CuV, and B19V NS1, respectively.

To identify mAbs recognizing NS1, we set up large-scale experiments to screen supernatants from hybridoma cells by indirect immunofluorescence. This approach has the advantage of directly identifying reactive antibodies and assessing their sensitivity and specificity for native proteins. Different strategies for the detection of NS1-specific Abs are illustrated in Figure 1C. To screen for antibodies recognizing rodent PVs we were able to rely on the genuine detection of NS1 produced after H-1PV infection in permissive cells in comparison to non-infected counterparts. This was exemplified (framed red) with hybridoma supernatants producing the previously established mAb 3D9 [18,26], demonstrating the specific detection of NS1 even at lower concentrations (i.e., 1:5 dilution). Since virus/host cell systems to produce NS1 from genuine CuV and B19V infections were not available, we searched for alternative approaches. Recombinant baculoviruses are easily established, capable of expressing large amounts of functional NS1 proteins under the control of the polyhedrin promoter in insect cells, and suitable for purification as well as in vitro characterization [27]. Thus, we generated recombinant baculoviruses expressing CuV NS1 with N-terminal signal sequences (rBac-yCutaNS1 and rBac-FlagCutaNS1, respectively), allowing us to monitor the expression of the recombinant proteins. The suitability of this screening approach was then tested with rBacH1~NS1 expressing H-1PV NS1 using previously established mAbs (this work) and (N-terminal-tagged) CuV NS1 using polyclonal anti-YDGASS rabbit serum, raised to identify the minor form of MVM NS2 [18], or commercially available monoclonal anti-Flag antibodies (framed blue). Although with this system background fluorescence was higher, it allowed us to screen for CuV NS1 antibodies. As an alternate possibility, we considered the use of recombinant vaccinia viruses. This system was used earlier to study the nuclear localization and oligomerization of MVM NS1 by means of a co-transport assay [28], and was proven to produce correctly phosphorylated and functional NS1 polypeptides [29,30,31]. Similarly, recombinant vaccinia viruses, expressing H-1PV, CuV, BuV, and B19V NS1 under the control of the bacteriophage T7 promoter, were generated, and the parvoviral proteins were expressed through co-infection with vTF7-3, another recombinant vaccinia virus providing T7 polymerase [32]. The suitability of this approach was established with vvH1-NS1 followed by detection with mAB 3D9 and vvB19-NS1, detected with the polyclonal serum αNS1_H_, respectively (framed brown).

### 2.2. Identification of Hybridoma Producing mAbs for NS1 Detection

#### 2.2.1. H-1PV NS1

**Figure 2:**
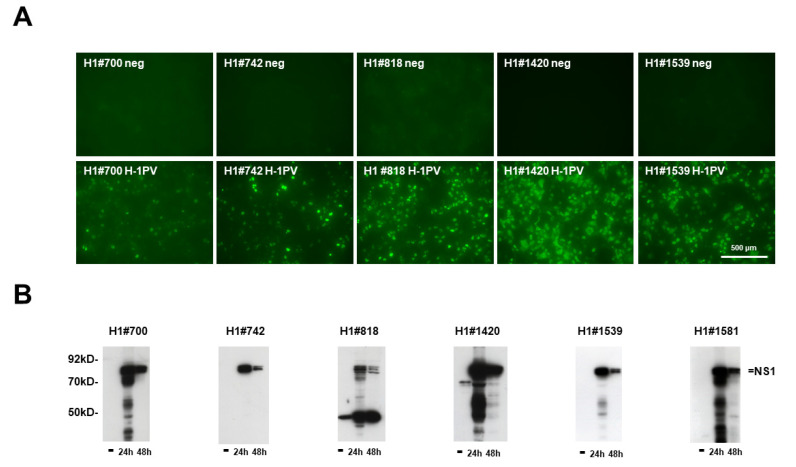
Screening for suitable mAbs that recognize rodent H-1PV NS1. (**A**) Detection of H1-NS1 by indirect immunofluorescence with candidate hybridoma supernatants. NBK cells grown in 96-well plates were infected (or not) with H-1PV, fixed with formaldehyde at 24 h p.i., and analyzed with candidate hybridoma supernatants containing primary antibodies, followed by Alexa-Fluor488-conjugated goat anti-mouse IgG. Pictures were taken with a Keyence3000 microscope at 10-fold magnification. Scale bar represents 500 μm. (**B**) Western blot detection of H1-NS1 with candidate hybridoma supernatants. NBK cells were infected (or not) with H-1PV and harvested at 24 and 48 h p.i., respectively. Cellular extracts were separated by 10% SDS-PAGE, blotted on nitrocellulose membranes, and treated with hybridoma supernatants. Detection was performed with HRP-conjugated goat anti-mouse IGs, followed by ECL detection and X-ray films. Molecular weights and the position of NS1 are indicated.

Although rodent protoparvovirus NS1 proteins are rather conserved among the genus, there are also significant variations, particularly in regions that potentially interact with host cell proteins [33]. In order to obtain mAbs with optimal reactivity but different characteristics, we characterized roughly 250 IF-positive seed clones for their specificity by indirect immunofluorescence and Western blot (Figure 2; Table 1). Among these mAbs, 90 scored “excellent” for specificity and sensitivity, 101 “intermediate”, and only 62 showed “poor” or significant non-specific reactivity in non-infected control cells. In addition, we tested 225 of these supernatants in Western blots to detect denatured NS1 in cellular extracts. Again, 32 supernatants proved to contain mAbs with strong and specific reactivity, while 50 scored weaker and/or detected non-specific band(s) in non-infected control extracts, while 94 scored poor and 49 showed no reactivity at all. Interestingly, as exemplified with H1#818 (Figure 2), among the 90 mAbs with high sensitivity and specificity in IFs roughly 50% performed rather poorly in Western blot, demonstrating weak (14) or even no (26) detectable reactivity for denatured NS1. Conversely, four mAbs that performed poorly in IFs showed excellent qualities in WBs (Table 1). These findings strongly suggest that, besides the linear amino acid sequence, conformation could be an important determinant in the detection of native NS1 by immunolocalization. A number of seed clones with “excellent” or “intermediate” reactivity in immunofluorescence were saved, selected for subcloning, and used for further testing, such as the specificity for H-1PV NS1 and strongly related species (e.g., KRV (Kilham rat parvovirus) versus mAbs capable of detecting the NS1 of more distinct species (LuIII, MVM, and TVX (tumor virus X)).

#### 2.2.2. CuV NS1

**Figure 3:**
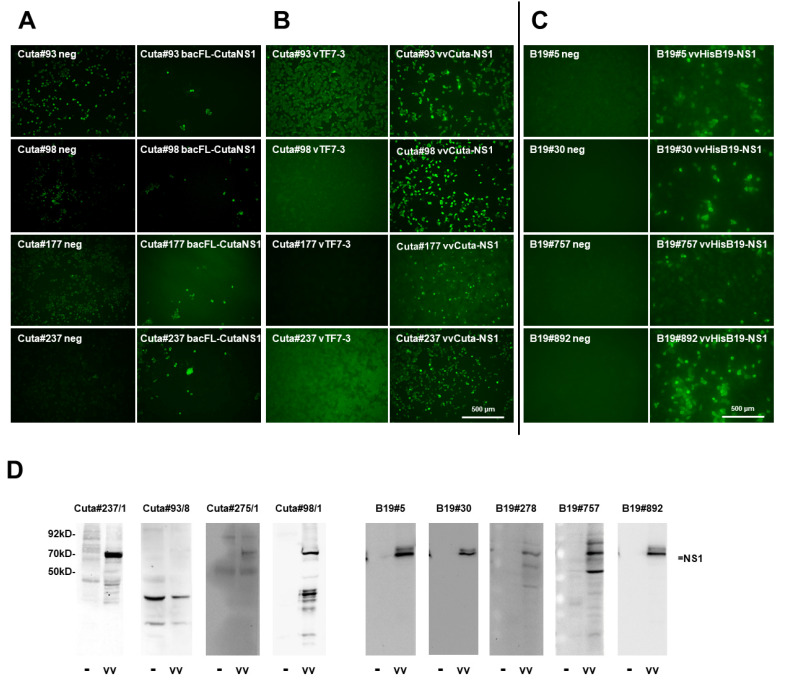
Screening for suitable mAbs for recognizing CuV and B19V NS1, respectively. (**A**) Examples for IF screening using Sf-9 cells infected (or not; neg) with recombinant baculoviruses expressing CuV NS1 (bacFL-CutaNS1), under the control of the polyhedrin promoter. Sf-9 cells grown in 96-well plates were infected (or not) with bacFL-CutaNS1, fixed with formaldehyde at 48 h p.i., and analyzed with candidate hybridoma supernatants containing primary antibodies, followed by Alexa-Fluor488-conjugated goat anti-mouse IgG. Pictures were taken with a Keyence3000 microscope at 10-fold magnification. Scale bar represents 500 μm. (**B**) HeLa cells grown in 96-well plates were infected (or not) with recombinant vaccinia viruses vTF7-3 (providing the T7 polymerase) (left) or vTF7-3+vvCutaNS1 (right), fixed after 24 h with formaldehyde, and NS1 was detected with the indicated hybridoma supernatants and Alexa-Fluor488-conjugated goat anti-mouse IgG. Pictures were taken with a Keyence3000 microscope at 10-fold magnification. Scale bar represents 500 μm. (**C**) HeLa cells grown in 96-well plates were infected (or not) with recombinant vaccinia viruses vTF7-3+vvHisB19-NS1, fixed after 24 h with formaldehyde, and B19-NS1 was detected with candidate hybridoma supernatants and Alexa-Fluor488-conjugated goat anti-mouse IgG. Pictures were taken with a Keyence3000 microscope at 10-fold magnification. Scale bar represents 500 μm. (**D**) Western blot detection of Cuta-NS1 and B19-NS1, respectively, with candidate hybridoma supernatants. HeLa cells were infected (vv) or not (−) with vTF7-3+vvCuta-NS1 (left) or vvB19-NS1 (right), respectively, and harvested at 24 h p.i. Cellular extracts were separated by 10% SDS-PAGE, blotted on nitrocellulose membranes, and treated with hybridoma supernatants. Detection was performed with HRP-conjugated goat anti-mouse IGs, followed by ECL detection using INTAS imaging systems or X-ray films. Molecular weights and the position of NS1 are indicated.

The screening for mAbs to detect CuV NS1, expressed by means of recombinant baculoviruses in Sf-9 insect cells, proved to be significantly less reliable than expected with the use of established reagents (Figure 1B). Thus, fluorescence intensity between NS1-producing versus non-infected cells was often minute, and the subcellular distribution of the viral protein was not indicative for a distinct detection of the antibody with CuV NS1 (Figure 3A). We decided to keep 16 apparently positive seed clones to be further evaluated with an alternative screening approach, using recombinant vaccinia viruses to express NS1 in mammalian cells. This follow-up screening delivered six positive clones by IF, of which three out of four showed CuV NS1 in Western blots (Figure 3B,D and Table 1).

#### 2.2.3. B19V NS1

To identify mAbs for recognizing B19-NS1, we used recVV to express the viral protein in HeLa cells. As shown in Figure 3C,D, with this screening approach we obtained 10 IF-positive clones, of which all five clones analyzed by Western blot analyses were able to detect B19-NS1 as a denatured polypeptide in cellular extracts (Table 1).

### 2.3. Assessment of Antibody Cross-Reactivity between Rodent and Human Parvoviruses

**Figure 4:**
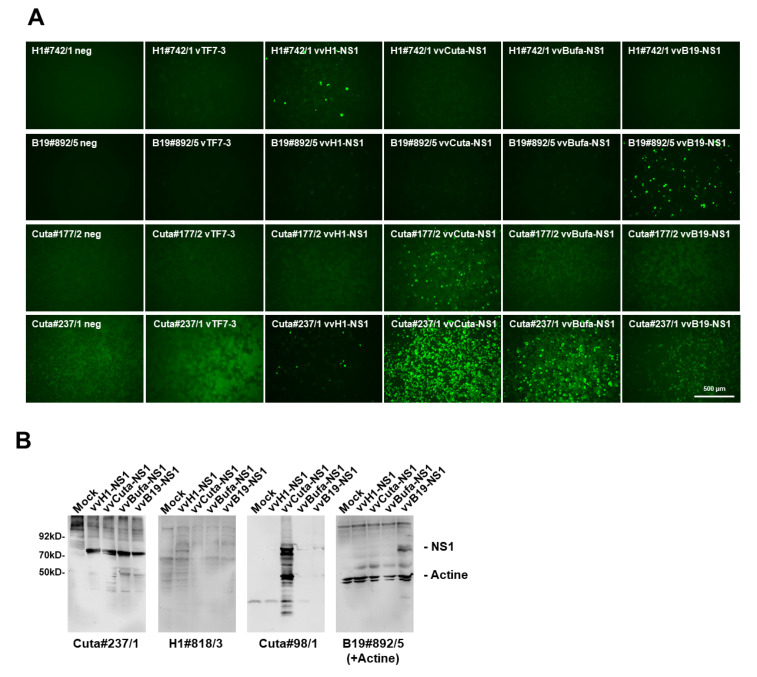
Specificity versus cross-reactivity of selected mAbs regarding rodent and human PV-NS1, respectively. (**A**) Hela cells grown in 96-well plates were infected (or not) with vTF7-3 alone, vTF7-3+vvH1-NS1, vvCutaNS1, vvBufaNS1, or vvB19-NS1, then were fixed with formaldehyde at 24 h p.i. and subjected to indirect fluorescence using the indicated hybridoma supernatants and Alexa-Fluor488-conjugated goat anti-mouse IgG. Pictures were taken with a Keyence3000 microscope at 10-fold magnification. Scale bar represents 500 μm. (**B**) Western blot detection of H-1PV-, CuV-, BuV-, and B19V-NS1, respectively, with candidate hybridoma supernatants. HeLa cells were infected (or not) with vTF7-3+vvH1-NS1, vvCuta-NS1, vvBufa-NS1, or vvB19-NS1, respectively, and harvested at 24 h p.i. Cellular extracts were separated by 10% SDS-PAGE, blotted on nitrocellulose membranes, and treated with hybridoma supernatants. Detection was performed with HRP-conjugated goat anti-mouse IGs, followed by ECL detection using the INTAS imaging system. Molecular weights and the positions of NS1 and actin are indicated.

Depending on the research question, it is not only important to unambiguously identify the presence of parvovirus activity in infected cells and tissues but also to eventually discriminate between potential human pathogen(s) and therapeutic rodent PVs used in cancer gene therapy. To determine the host range specificity versus cross-reactivity with the NS1 of other PV species, we took advantage of the vaccinia virus expression system. Indeed, recVV allow the production of post-translationally modified and functionally active NS1 polypeptides that closely resembled the virus-produced polypeptides [29]. This provided an excellent platform to compare the specificity/cross-reactivity of different mAbs towards polypeptides from different species, irrespective of their potential host range. We therefore expressed H-1PV, CuV, BuV, or B19V NS1 proteins, respectively, by means of recombinant vaccinia viruses in HeLa cells. Non-infected HeLa cells served as a negative control, and vTF7-3-infected HeLa cells served as a control for potential cross-reactivity with non-related viral proteins. Previously established polyclonal antisera NS1_H_ [23] and MK3/SP8 [24], the mAb NS1-3D9 [18,26], and a mAb isolated accidentally from a PV-infected mouse, m#236/10, served for comparisons. Indeed, as shown in Figure 4 and Figure S2, and summarized in Table 2, we could identify mAbs with different specificities, ranging from antibodies that were specific for rodent PV-NS1 to human protoparvovirus, or B19V NS1, respectively, but also some with a very broad PV host range, such as C#237/1, and others which, unfortunately, were able to detect vaccinia virus proteins (e.g., H1#700/6 or B19#278/3). This is in agreement with results obtained with polyclonal antisera NS1_H_ and NS1-MK/SP8, as well as Western blot experiments with selected candidate mAbs, which on the one hand detected all PV-NS1 (NS1_H_; C#237/1) while others were restricted to detecting NS1 species-specifically (NS1-MK3/SP8; m#236/10; H1#818/3; C#98/1; and B19#892/5).

**Figure 5:**
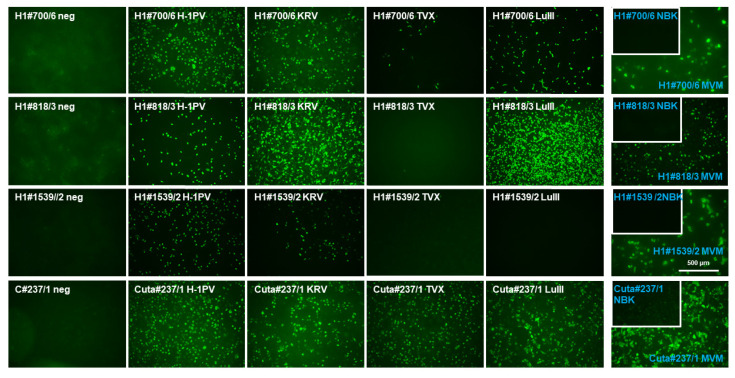
Cross-reactivity of selected mAbs regarding the NS1 of different rodent protoparvovirus species. HeLa (white) or NBK (blue) cells grown in 96-well plates were infected (or not, neg.) with the indicated protoparvoviruses: H-1PV [34], KRV (Isolate 1968 provided by G. Siegl), TVX (Isolate 1970 provided by G. Siegl), LuIII [35], or MVMp (MVM) [36]. After 24 h p.i., cells were fixed using formaldehyde and subjected to indirect fluorescence using the indicated hybridoma supernatants and Alexa-Fluor488-conjugated goat anti-mouse IgG. Pictures were taken with a Keyence3000 microscope at 10-fold magnification. Scale bar represents 500 μm.

We next examined those mAbs which were able to detect H-1PV NS1, assessing whether they had the capability to detect the different rodent protoparvovirus strains, i.e., possessed cross-reactivity with only closely related or more distant PV-NS1. Thus, HeLa and NBK cells, according to the respective host range, were infected (or not) with H-1PV, KRV, LuIII, TVX, and MVM, respectively, and subjected to immunofluorescence experiments using hybridoma supernatants obtained from either H-1PV or CuV NS1 immunizations. No specific signals were obtained with B19V-NS1-derived hybridoma. As shown in Figure 5 and Figure S3, and summarized in Table 2, two mAbs derived from CuV NS1 immunization (C#93/8, C#237/1) were able to detect all five rodent PV-NS1, while H-1PV NS1 immunization produced a panel of mAbs with different host range spectra: cross-reactivity with all five NS1 species (e.g., H1#700/6), H-1PV, KRV, LuIII, MVM (e.g., H1#818/3), H1-PV, KRV, and MVM (e.g., H1#1539/2), or H-1PV, KRV, LuIII, and TVX (e.g., H1#705). This is contrary to the results obtained for both of the polyclonal antisera, which were unable to discriminate between rodent PV species.

### 2.4. Monoclonal Antibodies Detecting the C-Terminal Part of NS1

**Figure 6:**
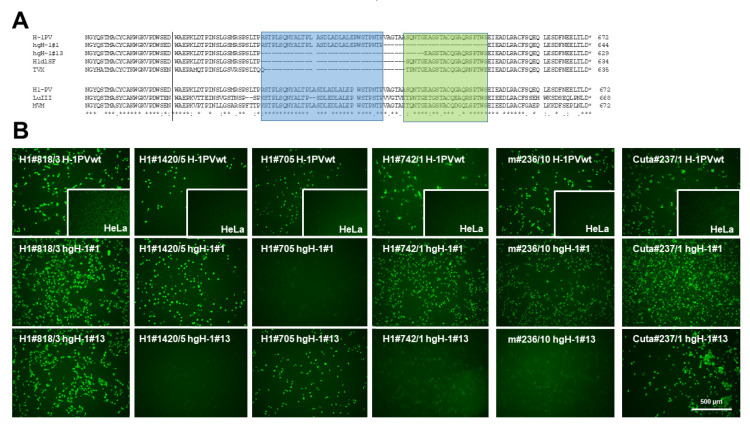
Localization of mAb-binding epitopes to the C-terminal NS1 domain. (**A**) Localization of *in-frame* deletions determined for TVX [37] and the H-1PV variants H1dlSF [38], hgH-1#1, and hgH-1#13 [39], respectively, are shown together with the wild-type H-1PV NS1 amino acid sequence on the top. The bottom presents an alignment between H-1PV, LuIII, and TVX NS1. Identical aa are denoted with (*) and conserved substitutions with (:) or (.). A potential binding area of mAbs that fail to detect hgH-1#13 is indicated in blue, and of H1#705 that fails to detect hgH-1#1 in green. The amino acid numbers of the respective NS1 species are denoted on the right. (**B**) Assessment of mAb binding to hgH-1#1 and hgH-1#13 NS1, respectively, as compared to the wild-type polypeptide was by indirect immunofluorescence. HeLa cells grown in 96-well plates were infected (or not, insert) with the indicated H-1PV variant, fixed at 24 h p.i., and NS1 detection was assessed for different mAbs using the indicated hybridoma supernatants and Alexa-Fluor488-conjugated goat anti-mouse IgG. Pictures were taken with a Keyence3000 microscope at 10-fold magnification. Scale bar represents 500 μm.

**Figure 7:**
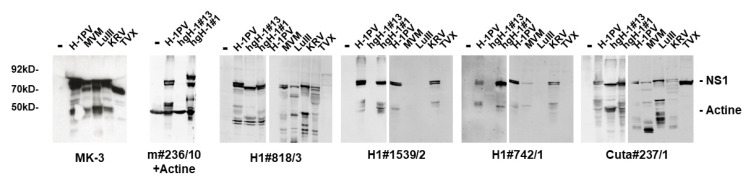
Detection of rodent protoparvovirus NS1 variants by Western blotting. HeLa or NBK cells were infected (or not) with the indicated protoparvoviruses: H-1PV [34], hgH-1#1, and hgH-1#13 [39]; KRV (Isolate 1968 provided by G. Siegl), TVX (Isolate 1970 provided by G. Siegl), LuIII [35], or MVMp [36]. Cells were harvested at 24 h p.i. and cellular extracts were separated by 10% SDS-PAGE, blotted on nitrocellulose, and subjected to detection of NS1 using the indicated hybridoma supernatants. Rabbit NS1-MK3/SP8 served as a positive control for the different PV strains. Detection was performed with HRP-conjugated goat anti-mouse IGs, followed by ECL detection using the INTAS imaging system. Molecular weights and the positions of NS1 and actine are indicated.

Interestingly, a significant number (6/19) of H-1PV-NS1-specific mAbs, including the clone m#236/10, that showed cross-reactivity with NS1 from different rodent PV species (i.e., KRV, LuIII, and MVM) completely failed to detect TVX NS1 by indirect immunofluorescence. This is of particular interest since TVX was recognized by an in-frame deletion in the C-terminal region of NS1/middle exon of NS2, similar to an H-1PV variant described by Faisst et al. [37,38], providing us with the ability to potentially localize a recognition epitope in NS1 for some mAbs. Besides the previously published deletions of Faisst’s DelH1 (H1dlSF) and TVX, we have identified two additional in-frame deletions of H-1PV variants derived after serial passaging in human glioblastoma cell lines hgH-1#1 and hgH-1#13 [39]. This lack of potential recognition epitopes in the context of H-1PV NS1 could allow us to identify mAbs that bind within these regions, irrespective of potential minor sequence alterations. Conversely, their failure to recognize deleted NS1 polypeptides might be indicative of the need to use alternative reagents. Figure 6A depicts the location of the two deletion variants under investigation, together with H1dlSF and TVX, as well as an alignment of H-1PV NS1 with LuIII and MVM covering this region. Indeed, as shown in Figure 6B and Table 2, a large proportion of the mAbs tested detected the wild type and both NS1 deletion mutants. However, one (H1#705) failed to detect hgH-1#1, and eight mAbs, including m#236/10, failed to detect hgH-1#13, of which three of them, m#236/10, H1#1539/2, and H1#742/1, also failed to detect the denatured polypeptide in Western blots (Figure 7). This strongly suggests that these antibodies interact with the C-terminal part of NS1, and especially recognize an area of NS1 that is subject to deletion in a host-range-dependent manner. In addition, we tested some mAbs for specificity/cross-reactivity for fully denatured NS1 proteins by Western blotting. Indeed, Cuta#237/1 was capable of detecting all protoparvovirus NS1 in Western blots, while #818/3 failed to detect CuV and TVX NS1. As for H1#1539/2 and H1#742/1, which both did not detect hgH-1#13, it is of interest to note that both were able to detect MVM-NS1 under native conditions, but only H1#742/1 interacted with the denatured polypeptide, while H1#1539/2 did not.

### 2.5. Monoclonal Antibodies Detecting H-1PV in Paraffin-Embedded Samples

**Figure 8:**
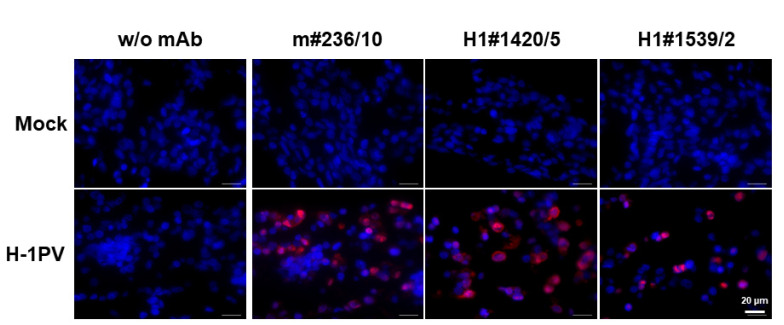
Detection of H-1PV NS1 in slices derived from paraffin-embedded cell blocks. NBK cells grown in tissue culture were infected (or not, “mock”) with H-1PV, harvested at 24 h p.i., and embedded into paraffin. Three affinity-purified IgGs were tested for their capacity to detect, based on C-terminal region recognition, H-1PV NS1. Highly specific NS1 detection with no background staining was achieved by all three mAbs tested, demonstrating their reactivity on paraffin slices. Blue: DAPI; red: cyanine 3 (Cy3). Scale bar: 20 μm.

Some laboratory research and clinical applications require immunohistochemical analyses of paraffin-embedded tissues. In order to test whether some of our H-1PV NS1 mAbs were able to specifically detect NS1 in paraffin-embedded sections, we used the cell block approach for sample preparation. The latter mimics the histological processing of tissues and allows cell cultures to be used as controls for tissue immunohistochemical analyses. As illustrated in Figure 8, at least three of the mAbs tested, namely m#236/10, H1#1420/5, and 1539/2, specifically detected H-1PV NS1 in paraffin-embedded cell blocks prepared from H-1PV-infected cells (lower panels). Non-specific mAb binding was not detected, as demonstrated by the lack of background immunofluorescence in the control cell blocks obtained from non-infected cells (upper panels).

## 3. Discussion

To determine and analyze viral infections in vivo, appropriate sensitive and specific tools are required. Apart from molecular biological methods, such as quantitative PCR to monitor the presence of viral DNA in tissues, blood, and/or body fluids, as well as RT-PCR to assess the presence of RNA transcripts determining viral activity in susceptible cells, it is valuable to detect viral proteins with defined functions in the target cell by immunohistochemistry. For instance, co-localization studies with cellular target proteins were able to provide important information regarding the replication process [12], virus propagation and spreading [15,40], and the potential impact/damage of the virus to the target tissue [40]. In particular, such details are of importance for the understanding and follow-up of chronic infections in affected tissues [8], and rating of novel therapeutic approaches, such as cancer therapy using oncolytic viruses by monitoring the benefit of the treatment and assessing potential efficacy [41,42]. To obtain such possibilities, it is necessary to generate suitable (monoclonal) antibodies that can recognize the native target protein(s) with high sensitivity and specificity.

Due to the small coding capacity of PVs, the amount of viral target proteins required to monitor infections is very limiting. Indeed, most viral activities are driven by a single multifunctional protein, NS1, which is regulated through the infection process by differential phosphorylations, enabling this polypeptide to exert distinct enzymatic activities and interact with multiple different proteins [17,25,33]. The other, even smaller non-structural proteins, although necessary for productive infections, merely assist this polypeptide to execute processes in a concerted way [25]. Thus, the presence of NS1 early, as well as late, in infection, and its rather long half-life, makes this viral protein an ideal candidate to monitor both productive and abortive PV infections in cells and tissues. This is not only the case for rodent parvoviruses but appears to be a general feature for *Parvovirinae* NS1 proteins, since B19V NS1 plays a similar key role during infection, also significantly differing in its primary sequence and ability to interact with host cell proteins and processes [7,19].

Ideally, it is desirable to establish tools that are not only able to specifically recognize a distinct viral protein of a single species but can be used for related viruses as well. This is particularly the case for a class of emerging viruses, such as the human protoparvoviruses, for which three new viruses have been identified in the last five years [9,43,44]. On the other hand, it might also be desirable to generate a panel of monoclonal antibodies with different properties, not only enabling the use of these agents in different approaches but also potentially providing the ability to discriminate between different virus species. In view of these possibilities, we generated antigens covering different regions of the polypeptide, considering areas that are highly conserved, such as the helicase domain, together with the most variable part of parvovirus NS1, the C-terminus, which is a region involved in both promoter regulation and protein/protein interactions at different stages of infection [19,33]. This strategy indeed proved successful, delivering highly specific mAbs that recognized NS1 only from closely related PV species, while others (e.g., Cuta#237/1) were recognized by their cross-reactivity with NS1 from more distant species.

To obtain optimal tools for distinct applications it is necessary to choose a screening system that provides the target protein in a suitable set-up. For our objective, obtaining mAbs that interact with the native, potentially post-translationally modified protein, we decided to use indirect immunofluorescence rather than Western blot. Indeed, our extensive comparison of seed clones by IF and WB (Table 1) clearly demonstrated that optimal mAbs for IFs potentially failed to detect NS1 in Western blot. This can be explained through the involvement of conformation aspects of the epitope, which might contribute significantly to the binding properties of the respective mAb. This is not without precedent, as determined by immunizations with small peptides (Figure S4). Indeed, the selection of phospho-specific mAbs that recognize P-135 of phospho-inositide-dependent kinase 1 (PDK-1) ([14]) delivered excellent mAbs that could recognize, specifically, phosphorylated PDK-1p135; however, they failed completely in WB and vice versa, strongly arguing for conformational aspects induced by this post-translational modification as an important determinant for the interactions of mAbs with the target epitope. Thus, it might be of importance to select a screening model that ensures, besides correct folding of the polypeptide, additional features, such as correct post-translational modifications.

To screen for mAbs with optimal reactivity, it is desirable to analyze the detection of native target proteins after genuine virus infections, and, as demonstrated with baculovirus expression of NS1 in insect cells, alternative expression systems might not deliver the desired results. However, particularly regarding emerging viruses, suitable virus/host cell systems are not always easily accessible. In addition, it might be useful to assess the cross-reactivity of viral proteins from different origins using the same conditions, i.e., the same host cells. In this regard, high-level expression of the polypeptides with recombinant vaccinia viruses in human cell lines appeared to be justified, since it was shown to generate functionally active and post-translationally properly modified NS1 [29,30,31]. Indeed, as exemplified using Cuta#237/1, which was proven to cross-react with rodent PV NS1, it is certainly feasible to obtain reliable tools using this heterologous expression system in mammalian cells.

Interestingly, recent investigations with potential oncolytic parvoviruses have identified *in-frame* deletions in the C-terminus of NS1 and the middle exon of NS2 [37]. TVX, which was isolated as an opportunistic infectant in human cancer cell lines [3], and an H-1PV variant, H1dlSF, were found upon cultivation in NBE cells, expressing an NS1 polypeptide harboring a deletion of 37 amino acids in the C-terminus [38]. Moreover, additional H-1PV NS1 variants derived after serial passaging in human glioma cell lines [39] were found to harbor similar deletions within this region. This region, prone to being mutated in a host-range-dependent manner, seems to be an excellent epitope for antibodies recognizing native NS1 by indirect immunofluorescence, unless affected through *in-frame* deletions. Thus, we concluded that this region of NS1 is exposed in the native protein and offers excellent epitopes not only for the binding of specific antibodies during the selection procedure but also for potential interactions with cellular targets, which are essential to drive the virus through the replication cycle [17]. Indeed, this exposed area of NS1 harbors phosphorylation sites that are targeted by cellular kinases late in infection [29,45], and is thought to be involved in the organization of viral egress and cell lysis [15,45]. Altogether, it is tempting to speculate on whether antibodies binding to this NS1 region could eventually inhibit specific interactions with host cell factors, consequently affect the progression of the infection cycle, and be used to determine specific NS1 functions necessary at late stages of infection.

In conclusion, the presented approach generated a panel of mAbs with different specificities towards PV species to detect and monitor parvovirus infections in tissue, including paraffin sections. This will allow us to study persistent and/or chronic infections of human parvoviruses as a potential disease-causing agent. We also generated tools to monitor the effects of a virotherapy of cancer, not only for H-1PV currently under investigation [46] but additionally for related candidates, such as LuIII and TVX. Indeed, these reagents might not only help to monitor infections after application in the target tumor tissue but also determine the suitability of the agent in a pre-treatment diagnosis, assessing permissiveness and efficacy after ex vivo infection of cultivated tumor tissues. However, such applications might be improved through combinations with additional antibodies that recognize progeny particle production and spreading.

## 4. Material and Methods

### 4.1. Animal Ethics Statement

Experiments on animals were conducted according to institutional and legal regulations for animal experimentation, as approved by the Animal Welfare Committee of the German Cancer Research Center and by the Land Baden-Württemberg Az.35-9185.82/A8-17.

### 4.2. DNA Constructs

Synthetic DNA: Cuta- (Accession KT868814.1 AMS35105.1) and Bufa-NS1 JQ918261.1 DNAs were obtained through DNA synthesis, cloned as PmeI to NcoI fragments into pFastBac1 from BioCat.

*Immunogens:* To obtain constructs to express H-1PV, B19V, and CuV immunogens in bacteria, the required PV sequences were generated by PCR using primer pairs containing appropriate restriction enzyme sequences to clone these sequences as *in-frame* insertions into pQE-32 (Qiagen). PCR was performed using a LongAmp PCR-KIT (NEB), using the primer pairs listed in Table 3, purified on agarose gels and cloned into pCR2.1 (Invitrogen). The fragments encoding the respective immunogens H1-NS1_Heli_, H1-NS1_TA_, B19-NS1_Heli_, B19-NS1_TA_, and CutaNS1_Im_ were cloned separately as BamHI/NotI fragments in a pQE-32 plasmid derivative, containing a NotI cleavage site downstream of BamHI.

*Plasmids for recombinant baculo- and vaccinia viruses:* Full-length NS1, Flag-, YDGASS- or His-tagged NS1 sequences were obtained by PCR using a LongAmp PCR-KIT (NEB), using the primer pairs listed in Table 3 to generate FL-H1~NS1, Y-CutaNS1, FL-CutaNS1, FL-B19NS1, and HisB19NS1, respectively, with appropriate restriction enzyme recognition sites flanking the coding sequences. PCR fragments were then cloned into pCR2.1, and for the generation of recombinant baculo viruses were cloned as PmeI/NotI fragments into StuI- and NotI-cleaved pFastBac1 (BioCat) to obtain pFastBac-H1-NS1, pFastBacY-CutaNS1, and pFastBacFL-CutaNS1. To generate recombinant vaccinia viruses, PmeI/XhoI-cleaved fragments were ligated into StuI- and XhoI-cleaved pTM-1 to obtain pTM1-H1-NS1, pTM1-FL-BufaNS1, pTM1-FL-CutaNS1, and pTM1-HisB19NS1, respectively.

### 4.3. Reagents

Primary antibodies and antisera: Antisera NS1_H_ [23] and NS1-MK3/SP8 [24], as well as monoclonal antibody 3D9 [18,26], were raised against bacterially expressed peptides corresponding to MVM-NS1 sequences. mAB m#236/10 was identified in a screen from an animal with reactivity towards a mouse PV-NS1. Anti-NS2_Y_ was generated with a KLH-coupled YDGASS peptide [18]. Monoclonal anti-Flag was purchased from SIGMA, and anti-actin from MP chemicals.

Secondary antibodies: Horseradish-peroxidase-conjugated anti-rabbit and anti-mouse IgGs were from Promega. All fluorescent-dye-labeled IgGs were from Invitrogen.

### 4.4. Cells and Viruses

*Cell lines:* HeLa, NBK, A9, CV-1, and BSC40 cells were maintained as monolayers in Dulbecco’s Modified Eagle’s Medium (DMEM, Gibco), while Sf9 cells were grown in suspension using TNM-FH insect medium (SIGMA) containing 10% fetal calf serum (FCS).

*Viruses:* Parvoviruses: MVMp (pMVMp) [36] was propagated in A9 cells; H-1PV (pSR19) [34], hgH1#1, and hgH1#13 H-1PV variants were selected for propagation in human glioma cells [39], LuIII [35] KRV68 (Isolate 1968) [3] in NBK, and TVX (Isolate 1970) [3] was propagated in HeLa. Baculoviruses: Recombinant baculoviruses were then generated using a Bac-To-Bac^TM^-KIT (Invitrogen, Waltham, MA, USA) and grown in Sf-9 cells. Expression of the respective recombinant proteins was ensured by indirect immunofluorescence using appropriate antibodies (H1-NS1: NS1-MK3/SP8; Y-CutaNS1: polyclonal peptide-antisera raised against pYDGASS, the C-terminus of the minor MVM-NS2 variant [18]; FL-CutaNS1 using anti-Flag mAbs (SIGMA, St. Louis, MO, USA)). Vaccinia: Recombinant vaccinia viruses were generated as described previously [32]. In brief: CV-1 cells were infected with VV-WR before transfection with the respective pTM1-NS1 construct using lipofectamine (Gibco), and were harvested after 48 h. Recombinant vaccinia viruses were then released by repeated freezing and thawing, and plaque purified in BSC-40 cells in the presence of BrdU. Individual plaques were then amplified in HeLa cells in the absence of BrdU and tested for the expression of the respective NS1 proteins in the presence of vTF7-3 [47], followed by indirect immunofluorescence using appropriate primary antibodies.

### 4.5. Bacterial Expression and Purification of Immunogens

Bacterial expression of peptides used for immunization was performed in E. coli SURE by induction with 1 mM IPTG for 24 h. Bacteria were then harvested by centrifugation at 2500 rpm, suspended in 400 ul Buffer 1 (Qiagen, Hilden, Germany) containing 10 mg/mL lysozyme, incubated for 20 min at room temperature, and treated with three cycles of freezing and thawing, before adding 1.6 mL TET (20 mM Tris, 10 mM EDTA, and 2% Triton X-100 pH 8.0) and 2 mL 2xRIPA (40 mM Tris, 10 mM EDTA, 2% Na-Deoxycholate, 2% NP-40, and 2% SDS pH 8.0) buffer. The suspension was then sonicated for 60 s and centrifuged at 8000 rpm for 20 min at 4 °C, and the pellet containing bacterial inclusion bodies was suspended in TE (10 mM Tris/1 mM EDTA pH 7.5). Proteins were then separated by SDS-PAGE, stained with Coomassie blue, and the protein bands corresponding to the immunogens were cut from the gel, eluted in 500 mM ammonium acetate, 10 mM MgAc, and 0.1% SDS overnight at 4 °C, before being dialyzed against PBS containing 20% sucrose and 10% glycerol.

### 4.6. Immunization, Cell Fusion, and the Isolation of Monoclonal Antibodies

For the generation of monoclonal antibodies, mice (mouse strain BalbC for H-1 or mouse strain C57black6N for Cuta and B19) from Jackson Immuno Research Laboratories, West Grove, PA, USA, were immunized via five subcutaneous injections into the hind leg with 20 μg recombinant bacterially expressed protein. To enhance the immune response, 100 μL of Freund’s Complete Adjuvant (Santa Cruz Biotechnology, Dallas, TX, USA) was added. Booster injections were conducted with Freund’s Incomplete Adjuvant, followed by injection with buffer only. Injections were given 3 to 8 days apart over up to a 30-day time period. Sero-positivity of peripheral blood samples of the animals was verified by Western blot. Lymph nodes of the popliteal fossa were isolated and lymphocytes were fused with Sp2/0 cells as described previously [48]. Seed clones producing specific antibodies were singularized by limiting dilutions into 96-well plates, in order to obtain 10 to 20 subclones derived from a single cell, and were further evaluated by immunofluorescence and Western blot. Up to three viable, antibody-producing colonies were then saved for additional studies. To purify mAbs from hybridoma supernatants, NS1-specific IgG were passed through 0.45 µm filters to eliminate non-soluble cell debris adjusted to 20 mM Na-phosphate pH 7.0 and loaded on a protein G-Sepharose column. After washing with 20 mM Na-phosphate pH 7.0 buffer, bound IgG were eluted with 0.1 M glycin-HCl pH 2.7 and dialyzed against phosphate-buffered saline pH 7.5.

### 4.7. Immunofluorescence Microscopy

Cells were grown in 96-well plates (Greiner), infected or not with the indicated viruses, fixed after 24 h with 3% formaldehyde, and permeabilized with 0.2% Triton X-100. The specimens were pre-adsorbed, incubated with primary antibodies, and stained with Alexa-Fluor488-conjugated anti-species antibodies. Analyses were performed with a Keyence microscope. Quantification of fluorescence intensity was performed using Fiji ImageJ software. The total fluorescent area was analyzed, integrated densities were measured, and fluorescence intensity (in relative fluorescence units, RFU) was determined as follows: negative, <20,000 RFU; (+), 20,000–30,000 RFU; (++), 30,000–40,000 RFU; and (+++), >40,000 RFU.

### 4.8. Western Blot Analysis

Cell extracts were produced by incubating cell pellets in an extraction buffer containing 20 mM Hepes-KOH pH 7.5, 300 mM NaCl, 1 mM EDTA, and 0.2% NP-40 on ice, and were clarified by centrifugation. Proteins were separated by SDS-PAGE, blotted onto nitrocellulose membranes, and identified with appropriate primary antibodies in 10% dry milk/PBS stained with horseradish-peroxidase-conjugated secondary antibodies for 1 h, and were detected by chemiluminescence (Amersham, UK). Protein bands were visualized in an ECL Chemocam (Intas Science Imaging Instruments GmbH, Göttingen, Germany).

### 4.9. Paraffin Embedment of Cultured Cells and Detection of Target Proteins

NBK cells were seeded in 150 mm × 25 mm cell culture dishes (Nunc). After subconfluent monolayer formation, cells were either infected with five plaque-forming units (PFU) of H-1PV or mock-treated with an equal volume of a virus-free cell culture medium. Then, 24 h after treatment, cells were scraped into the medium and centrifuged for 5 min/1000 rpm at room temperature. The pellet was washed with PBS, immobilized in agarose, and fixed overnight at room temperature in ROTI^®^ Histofix 4% (Carl Roth GmbH, Karlsruhe, Germany). The resulting cell blocks were further processed by paraffin embedding and sectioning using Leica HistoCore Arcadia H (Leica Biosystems, Tokyo, Japan) and a Microm HM 355S (Fisher Scientific, Rockingham County, NH, USA), respectively. Next, 10-µm-thick sections were stained with the purified mAbs, m#236/10, H1#1420/5, and H1#1539/2, specific for the H-1PV NS1 protein. Signals were visualized by secondary staining with a cyanine-3-conjugated anti-mouse antibody (115-165-003, Dianova, Hamburg, Germany). Fluorescence was assessed using a Zeiss Cell Observer Z1 and documented with ZEN blue edition software.

## Figures and Tables

**Table 1:** Summary reactive mAb clones in immunofluorescence (IF) and Western blot (WB).

**H-1PV NS1 Screening**					
IF-positive			WB-positive		
253			225		
Excellent ^1^	Intermediate ^2^	Weak ^3^	Excellent	Intermediate	Poor
90	101	62	32	50	94
**CuV NS1 Screening**					
IF-positive			WB-positive		
16			3		
**B19V NS1 Screening**					
IF-positive			WB-positive		
10			5		

^1^ (+++) > 40,000. RFU; ^2^ (++) 30,000–40,000 RFU; ^3^ (+) 20,000–30,000 RFU; and (neg) < 20,000 RFU.

**Table 2:** Cross-reactivity of individual mAbs.

Name	neg	vTF7-3	H1	Cuta	Bufa	B19	KRV	TVX	LuIII	MVM	Hg#1	Hg#13
**H-1PV-NS1-specific**												
H1#742/1	-	-	+++	-	-	-	+++	-	-	+++	+++	-
H1#1420/5	-	-	+++	-	-	-	+++	-	-	+++	+++	-
H1#700/6	-	++	+++	+++	+++	+++	+++	+++	+++	+++	+++	+++
H1#818/3	-	-	+++	-	-	-	+++	-	+++	+++	+++	+++
H1#1539/2	-	n.d.	+++	n.d.	n.d.	n.d.	+++	-	-	+++	+++	-
H1#705	-	n.d.	+++	n.d.	n.d.	n.d.	+++	+++	++	-	-	+++
H1#721	-	n.d.	+++	n.d.	n.d.	n.d.	+	-	++	++	+++	+++
H1#737	-	n.d.	+++	n.d.	n.d.	n.d.	n.d.	n.d.	n.d.	n.d.	+++	+++
H1#756	-	n.d.	+++	n.d.	n.d.	n.d.	n.d.	n.d.	n.d.	n.d.	+++	+++
H1#853	-	n.d.	+++	n.d.	n.d.	n.d.	++	-	+	++	+++	+++
H1#867	-	n.d.	+++	n.d.	n.d.	n.d.	+++	-	+++	+++	+++	+++
H1#888/1	-	n.d.	+++	n.d.	n.d.	n.d.	+++	-	+++	+++	+++	+++
H1#892/5	-	n.d.	+++	n.d.	n.d.	n.d.	+++	-	-	+++	+++	-
H1#905	-	n.d.	+++	n.d.	n.d.	n.d.	+++	-	-	+++	+++	-
H1#918	-	n.d.	+++	n.d.	n.d.	n.d.	+++	+++	+++	+++	+++	+++
H1#1252	-	n.d.	+++	n.d.	n.d.	n.d.	+++	-	-	++	+++	-
H1#1320	-	n.d.	+++	n.d.	n.d.	n.d.	+++	-	++	+++	+++	+++
H1#1428/5	-	n.d.	+++	n.d.	n.d.	n.d.	+++	-	-	(+)	+++	-
H1#1509/3	-	n.d.	+++	n.d.	n.d.	n.d.	+++	+++	+++	+++	+++	+++
H1#1581/3	-	n.d.	+++	n.d.	n.d.	n.d.	+++	+++	-	+++	+++	+++
**B19-NS1-specific**												
B19#892/5	-	-	-	-	-	+++	n.d.	n.d.	n.d.	n.d.	n.d.	n.d.
B19#757/10	-	-	-	-	-	+++	n.d.	n.d.	n.d.	n.d.	n.d.	n.d.
B19#5/12/3	-	-	(+)	(+)	(+)	+++	n.d.	n.d.	n.d.	n.d.	n.d.	n.d.
B19#278/3	-	+++	+++	+++	+++	+++	n.d.	n.d.	n.d.	n.d.	n.d.	n.d.
**Cuta-NS1-specific**												
C#177/2	-	-	-	+++	-	-	n.d.	n.d.	n.d.	n.d.	n.d.	n.d.
C#98/1	-	-	-	+++	+	-	n.d.	n.d.	n.d.	n.d.	n.d.	n.d.
C#93/8	-	+	+	+++	+++	+++	+++	+++	+++	+++	n.d.	n.d.
C#237/1	-	-	+++	+++	+++	++	+++	+++	+++	+++	+++	+++
**Others**												
pNS1_H_	-	-	+++	+++	+++	+++	+++	+++	+++	+++	n.d.	n.d.
pNS1-MK/SP8	-	-	+++	++	-	-	+++	+++	+++	+++	n.d.	n.d.
NS1-3D9	-	-	+++	-	-	-	+++	-	+++	+++	n.d.	n.d.
m#236/10	-	-	+++	-	-	-	+++	-	+++	+++	+++	-

(+++) > 40,000 RFU; (++) 30,000–40,000 RFU; (+) 20,000–30,000 RFU; (-), no reactivity, <20,000 RFU; and n.d. stands for not determined.

**Table 3:** Primer pairs used to generate PCR fragments.

	Forward Primer	Backward Primer
*Immunogens*		
H1-NS1_Heli_	*ggatccgcatggctagcaccagaacctgtagaatctttgc*	*gcggccgcttaaataactggtgttggttcaatctgtttgc*
H1-NS1_TA_	*ggatccgcatggcttgttactgtgctaaatggggcaaagt*	*gcggccgcttagtccaaggtcagctcctcgttgaagtcgc*
B19-NS1_Heli_	*gaaatttcctatgtgctgcttaaacaaacaaatgggaaaaag*	*gcggccgctattcgttggttgtcattatgactggtgttgg*
B19-NS1_TA_	*gtttaaacctatgggcgcctggaacactgaaaccccgcgctc*	*gcggccgcttactcataatctacaaagctttgcaatcc*
Cuta-NS1_Im_	*gaaatttcctatgtgctgcttaaacaaacaaatgggaaaaag*	*aagcttgcggccgcctaatagctggcattcacatccgttccggatatc*.
*Recombinant Viruses*		
FL-H1-NS1	*gtttaaacatggctgactacaaggacgacgatggctggaaacgcttactc*	*gcggccgcttactcataatctacaaagctttgcaatcc*
Y-CutaNS1	*gtttaaacatgagaccagagatcacgtggttgatggctctctcagcaaagagatg*	*aagcttgcggccgcctaatagctggcattcacatccgttccggatatc*
FL-CutaNS1	*gtttaaacatggctgactacaaggacgacgatggctctcagcaaagagatg*	*aagcttgcggccgcctaatagctggcattcacatccgttccggatatc*
FL-B19NS1	*gtttaaacatggctgactacaaggacgacgatggagctatttagaggggtg*	*gcggccgcttactcataatctacaaagctttgcaatcc*
HisB19NS1	*gtttaaacatgcactaccaccactaccac atggagctatttagaggggtg*	*gcggccgcttactcataatctacaaagctttgcaatcc*

## Data Availability

Not applicable.

## Supplementary Materials

The following are available online at https://www.mdpi.com/article/10.3390/pathogens11020208/s1, Figure S1: Assessment of conserved versus variable regions to generate suitable immunogens. Sequence alignments of H-1PV NS1 with polypeptides used for immunization to obtain mAbs that recognize CuV NS1 (A) and B19V NS1 (B). Sequence alignments were performed with EMBOSS Needle Pairwise Sequence Alignment (EMBL-EBI). The peptide sequences used as immunogens are indicated in red for CuV and B19V, respectively, and in green for H-1PV. (C) Determination of helical (red) and b-fold (blue) structures in the helicase domains of the three NS1 polypeptides, as determined by GAPPED-BLAST and PSI-BLAST [49]. Figure S2: Supplementary information to Figure 4: Specificity versus cross-reactivity of selected mAbs regarding rodent and human PV-NS1, respectively. HeLa cells grown in 96-well plates were infected (or not) with vTF7-3 alone, vTF7-3+vvH1-NS1, vvCutaNS1, vvBufaNS1, or vvB19-NS1, were fixed with formaldehyde at 24 h p.i., and were subjected to indirect fluorescence using the indicated hybridoma supernatants and Alex-Fluor488-conjugated anti-mouse-IgG. Pictures were taken with a Keyence3000 microscope at 10-fold magnification. Scale bar represents 500 µm. Figure S3: Supplementary information to Figure 5: Cross-reactivity of selected mAbs regarding NS1 of different rodent protoparvovirus species. HeLa (white) or NBK (blue) cells grown in 96-well plates were infected (or not, ‘neg’) with the indicated protoparvoviruses: H-1PV, KRV, TVX, LuIII, or MVMp were fixed with formaldehyde at 24 h p.i. and subjected to indirect fluorescence using the indicated hybridoma supernatants and Alex-Fluor488-conjugated anti-mouse-IgG. Pictures were taken with a Keyence3000 microscope at 10-fold magnification. Scale bar represents 500 µm. Figure S4: Binding properties of phospho-specific mAbs generated to detect PDKp135. (A) Peptide immunogen used to generate mAbs to specifically detect PDK-1(S135P). (B) PDK1-P135#202/2, a mAb suitable to detect PDK1-P135 in Western blots and indirect fluorescence. (C) PDK1-P135#12/6/1/1, a mAb suitable to detect PDK1-P135 as a native protein by IHC, but which failed to identify the denatured polypeptide in WB. (D) PDK1-P135#173, a mAb suitable to discriminate PDK1-P135 from the unmodified denatured polypeptide in WBs, but which performed poorly in IFs.
