# Supplementary material for: Generation and Validation of Monoclonal Antibodies Suitable for Detecting and Monitoring Parvovirus Infections
Source: Pathogens. 2022 Feb 4;11(2):208. doi: 10.3390/pathogens11020208 (PMC8877868; doi:10.3390/pathogens11020208)
Supplement: Supplementary file 1 [file pathogens-11-00208-s001.zip › mAb-NS1 Sup Figure S1.pptx]

## Slide 1
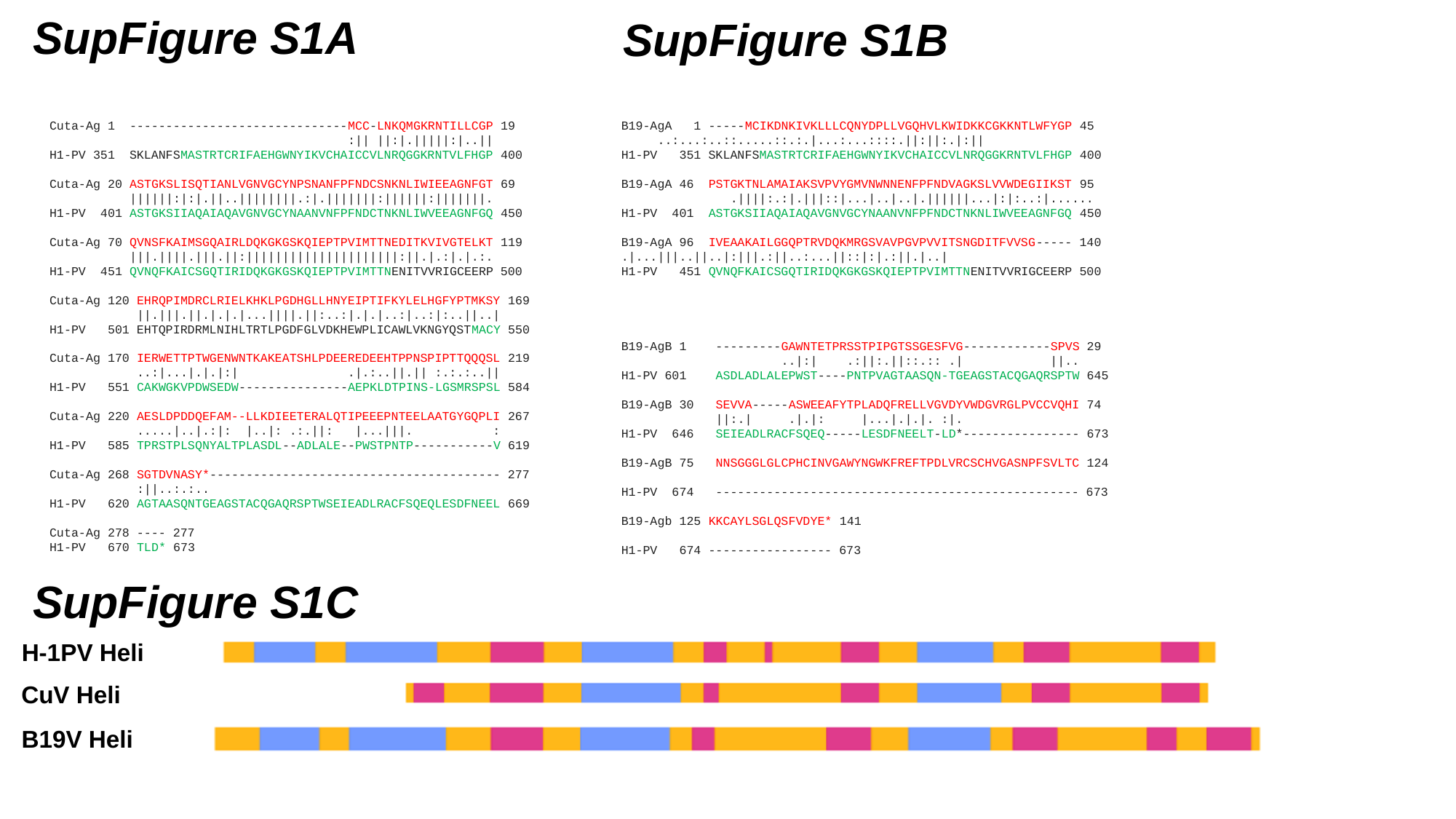

SupFigure S1A
SupFigure S1B
Cuta-Ag 1 ------------------------------MCC-LNKQMGKRNTILLCGP 19
		 :|| ||:|.|||||:|..||
H1-PV 351 SKLANFSMASTRTCRIFAEHGWNYIKVCHAICCVLNRQGGKRNTVLFHGP 400
Cuta-Ag 20 ASTGKSLISQTIANLVGNVGCYNPSNANFPFNDCSNKNLIWIEEAGNFGT 69
 ||||||:|:|.||..||||||||.:|.|||||||:||||||:|||||||.
H1-PV 401 ASTGKSIIAQAIAQAVGNVGCYNAANVNFPFNDCTNKNLIWVEEAGNFGQ 450
Cuta-Ag 70 QVNSFKAIMSGQAIRLDQKGKGSKQIEPTPVIMTTNEDITKVIVGTELKT 119
 |||.||||.|||.||:|||||||||||||||||||||:||.|.:|.|.:.
H1-PV 451 QVNQFKAICSGQTIRIDQKGKGSKQIEPTPVIMTTNENITVVRIGCEERP 500
Cuta-Ag 120 EHRQPIMDRCLRIELKHKLPGDHGLLHNYEIPTIFKYLELHGFYPTMKSY 169
 ||.|||.||.|.|.|...||||.||:..:|.|.|..:|..:|:..||..|
H1-PV 501 EHTQPIRDRMLNIHLTRTLPGDFGLVDKHEWPLICAWLVKNGYQSTMACY 550
Cuta-Ag 170 IERWETTPTWGENWNTKAKEATSHLPDEEREDEEHTPPNSPIPTTQQQSL 219
 ..:|...|.|.|:| .|.:..||.|| :.:.:..||
H1-PV 551 CAKWGKVPDWSEDW---------------AEPKLDTPINS-LGSMRSPSL 584
Cuta-Ag 220 AESLDPDDQEFAM--LLKDIEETERALQTIPEEEPNTEELAATGYGQPLI 267
 .....|..|.:|: |..|: .:.||: |...|||. :
H1-PV 585 TPRSTPLSQNYALTPLASDL--ADLALE--PWSTPNTP-----------V 619
Cuta-Ag 268 SGTDVNASY*---------------------------------------- 277
 :||..:.:..
H1-PV 620 AGTAASQNTGEAGSTACQGAQRSPTWSEIEADLRACFSQEQLESDFNEEL 669
Cuta-Ag 278 ---- 277
H1-PV 670 TLD* 673
B19-AgA 1 -----MCIKDNKIVKLLLCQNYDPLLVGQHVLKWIDKKCGKKNTLWFYGP 45 	 	 ..:...:..::.....::.:.|...:...::::.||:||:.|:||
H1-PV 351 SKLANFSMASTRTCRIFAEHGWNYIKVCHAICCVLNRQGGKRNTVLFHGP 400
B19-AgA 46 PSTGKTNLAMAIAKSVPVYGMVNWNNENFPFNDVAGKSLVVWDEGIIKST 95 	.||||:.:|.|||::|...|..|..|.||||||...|:|:..:|......
H1-PV 401 ASTGKSIIAQAIAQAVGNVGCYNAANVNFPFNDCTNKNLIWVEEAGNFGQ 450
B19-AgA 96 IVEAAKAILGGQPTRVDQKMRGSVAVPGVPVVITSNGDITFVVSG----- 140 	.|...|||..||..|:|||.:||..:...||::|:|.:||.|..|
H1-PV 451 QVNQFKAICSGQTIRIDQKGKGSKQIEPTPVIMTTNENITVVRIGCEERP 500
B19-AgB 1 ---------GAWNTETPRSSTPIPGTSSGESFVG------------SPVS 29
 ..|:| .:||:.||::.:: .| ||..
H1-PV 601 ASDLADLALEPWST----PNTPVAGTAASQN-TGEAGSTACQGAQRSPTW 645
B19-AgB 30 SEVVA-----ASWEEAFYTPLADQFRELLVGVDYVWDGVRGLPVCCVQHI 74
 ||:.| .|.|: |...|.|.|. :|.
H1-PV 646 SEIEADLRACFSQEQ-----LESDFNEELT-LD*---------------- 673
B19-AgB 75 NNSGGGLGLCPHCINVGAWYNGWKFREFTPDLVRCSCHVGASNPFSVLTC 124
H1-PV 674 -------------------------------------------------- 673
B19-Agb 125 KKCAYLSGLQSFVDYE* 141
H1-PV 674 ----------------- 673
SupFigure S1C
H-1PV Heli
CuV Heli
B19V Heli
