# Supplementary material for: Generation and Validation of Monoclonal Antibodies Suitable for Detecting and Monitoring Parvovirus Infections
Source: Pathogens. 2022 Feb 4;11(2):208. doi: 10.3390/pathogens11020208 (PMC8877868; doi:10.3390/pathogens11020208)
Supplement: Supplementary file 1 [file pathogens-11-00208-s001.zip › mAB-NS1 Sup Figure S2.pptx]

## Slide 1
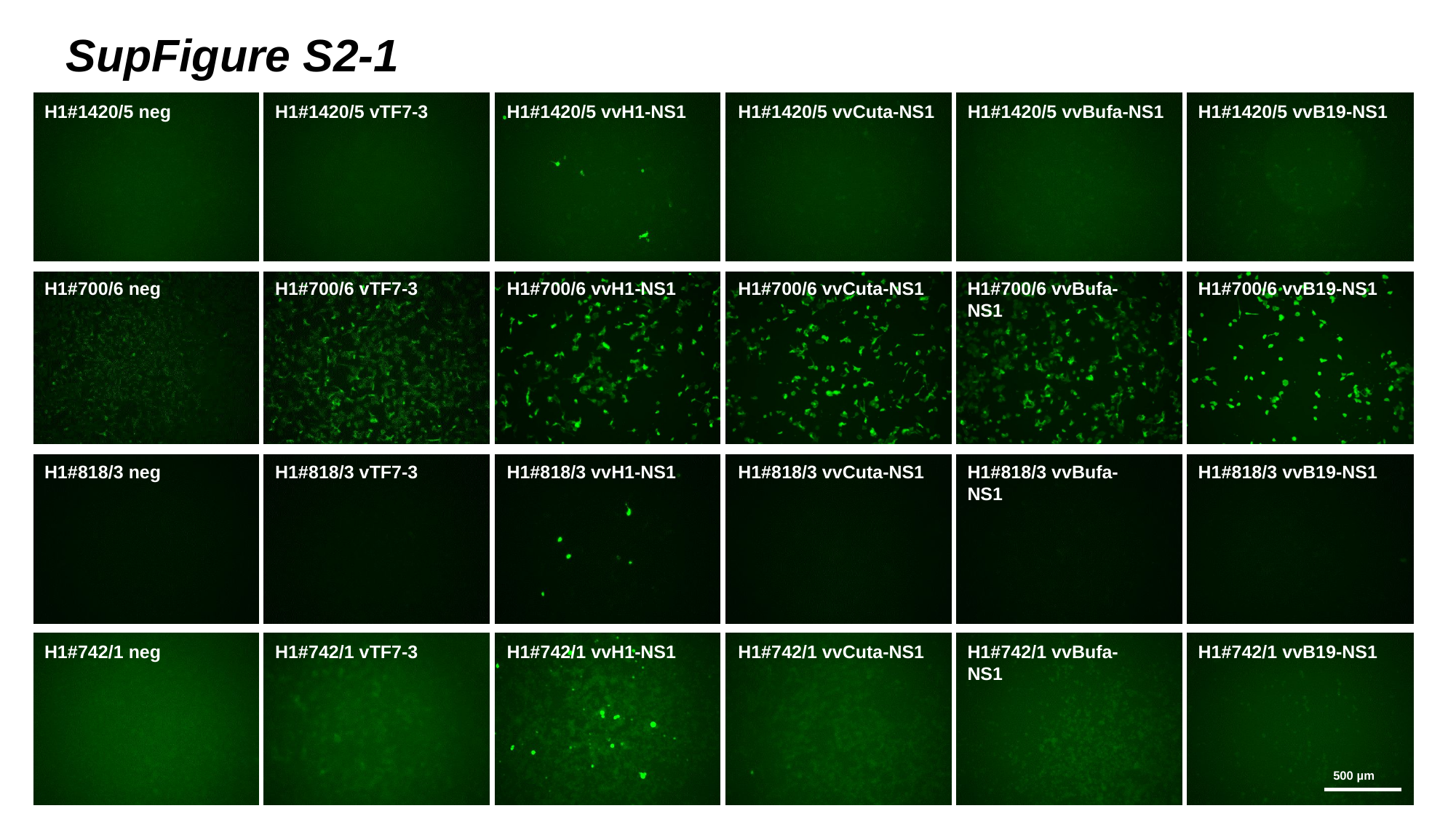

SupFigure S2-1
H1#1420/5 neg
H1#1420/5 vTF7-3
H1#1420/5 vvH1-NS1
H1#1420/5 vvCuta-NS1
H1#1420/5 vvBufa-NS1
H1#1420/5 vvB19-NS1
H1#700/6 neg
H1#700/6 vTF7-3
H1#700/6 vvH1-NS1
H1#700/6 vvCuta-NS1
H1#700/6 vvBufa-NS1
H1#700/6 vvB19-NS1
H1#818/3 neg
H1#818/3 vTF7-3
H1#818/3 vvH1-NS1
H1#818/3 vvCuta-NS1
H1#818/3 vvBufa-NS1
H1#818/3 vvB19-NS1
H1#742/1 neg
H1#742/1 vTF7-3
H1#742/1 vvH1-NS1
H1#742/1 vvCuta-NS1
H1#742/1 vvBufa-NS1
H1#742/1 vvB19-NS1
500 µm

## Slide 2
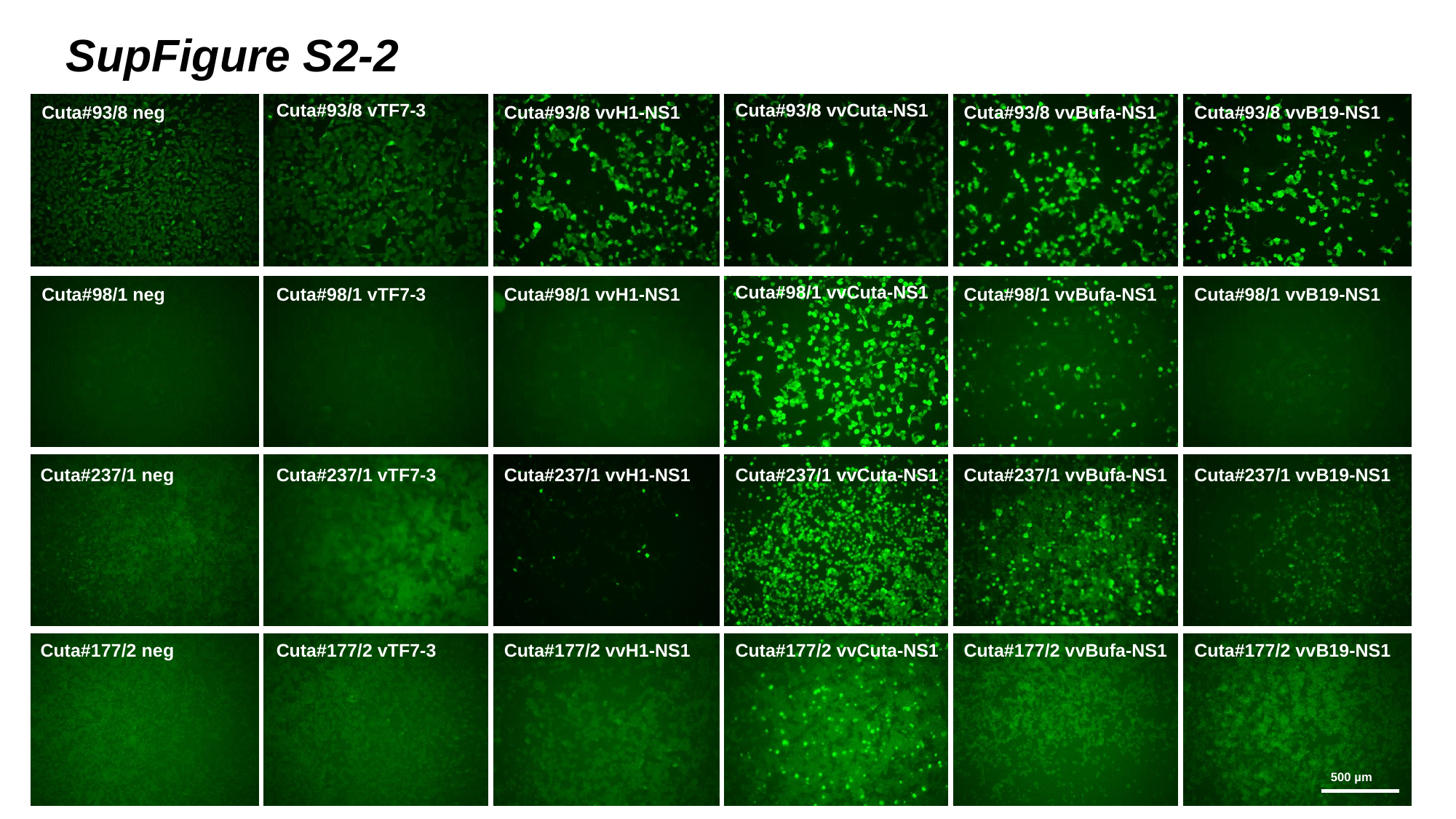

SupFigure S2-2
Cuta#93/8 vTF7-3
Cuta#93/8 vvCuta-NS1
Cuta#93/8 neg
Cuta#93/8 vvH1-NS1
Cuta#93/8 vvBufa-NS1
Cuta#93/8 vvB19-NS1
Cuta#98/1 vvCuta-NS1
Cuta#98/1 neg
Cuta#98/1 vTF7-3
Cuta#98/1 vvH1-NS1
Cuta#98/1 vvBufa-NS1
Cuta#98/1 vvB19-NS1
Cuta#237/1 neg
Cuta#237/1 vTF7-3
Cuta#237/1 vvH1-NS1
Cuta#237/1 vvCuta-NS1
Cuta#237/1 vvBufa-NS1
Cuta#237/1 vvB19-NS1
Cuta#177/2 neg
Cuta#177/2 vTF7-3
Cuta#177/2 vvH1-NS1
Cuta#177/2 vvCuta-NS1
Cuta#177/2 vvBufa-NS1
Cuta#177/2 vvB19-NS1
500 µm

## Slide 3
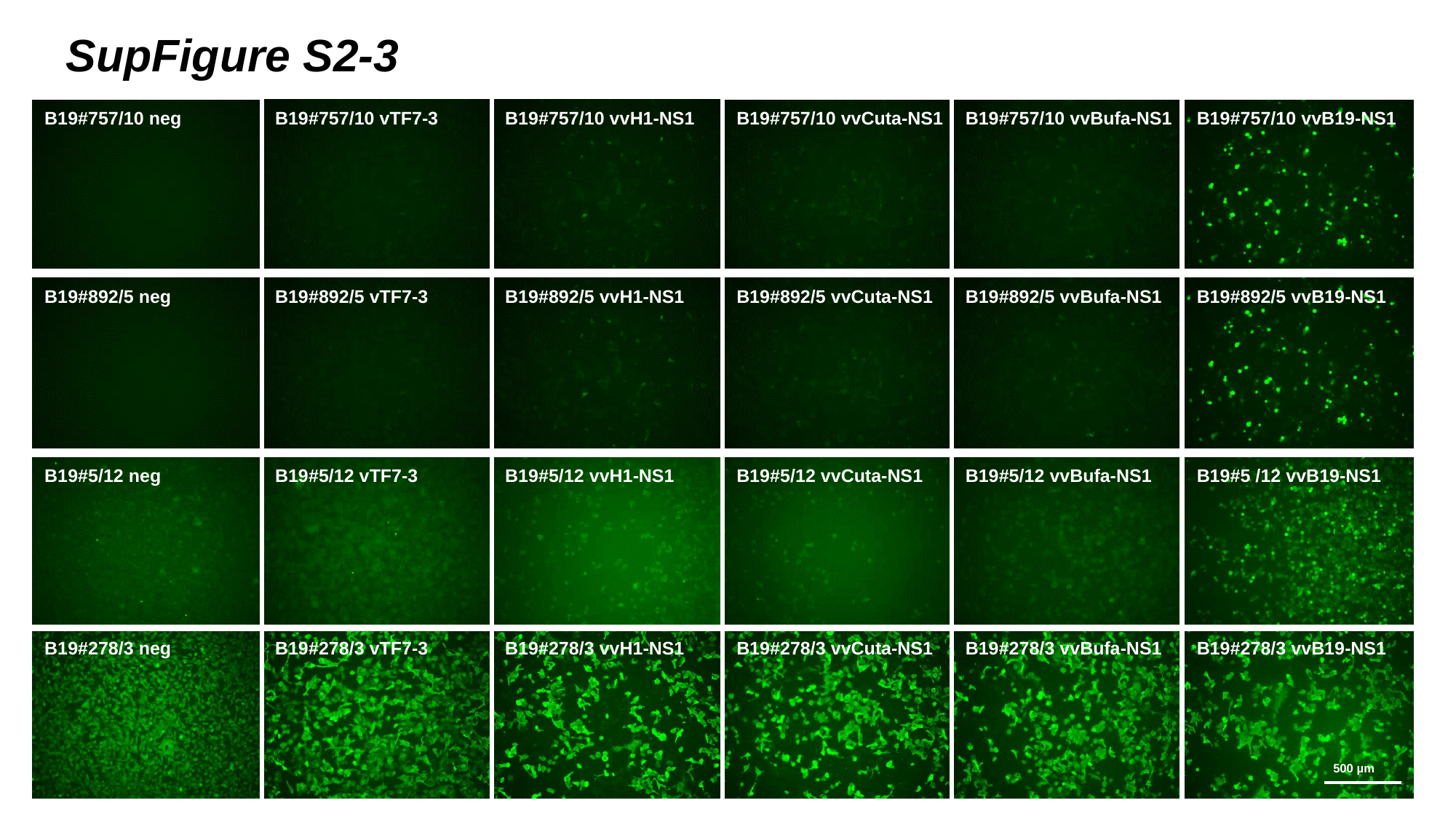

SupFigure S2-3
B19#757/10 neg
B19#757/10 vTF7-3
B19#757/10 vvH1-NS1
B19#757/10 vvCuta-NS1
B19#757/10 vvBufa-NS1
B19#757/10 vvB19-NS1
B19#892/5 neg
B19#892/5 vTF7-3
B19#892/5 vvH1-NS1
B19#892/5 vvCuta-NS1
B19#892/5 vvBufa-NS1
B19#892/5 vvB19-NS1
B19#5/12 neg
B19#5/12 vTF7-3
B19#5/12 vvH1-NS1
B19#5/12 vvCuta-NS1
B19#5/12 vvBufa-NS1
B19#5 /12 vvB19-NS1
B19#278/3 neg
B19#278/3 vTF7-3
B19#278/3 vvH1-NS1
B19#278/3 vvCuta-NS1
B19#278/3 vvBufa-NS1
B19#278/3 vvB19-NS1
500 µm

## Slide 4
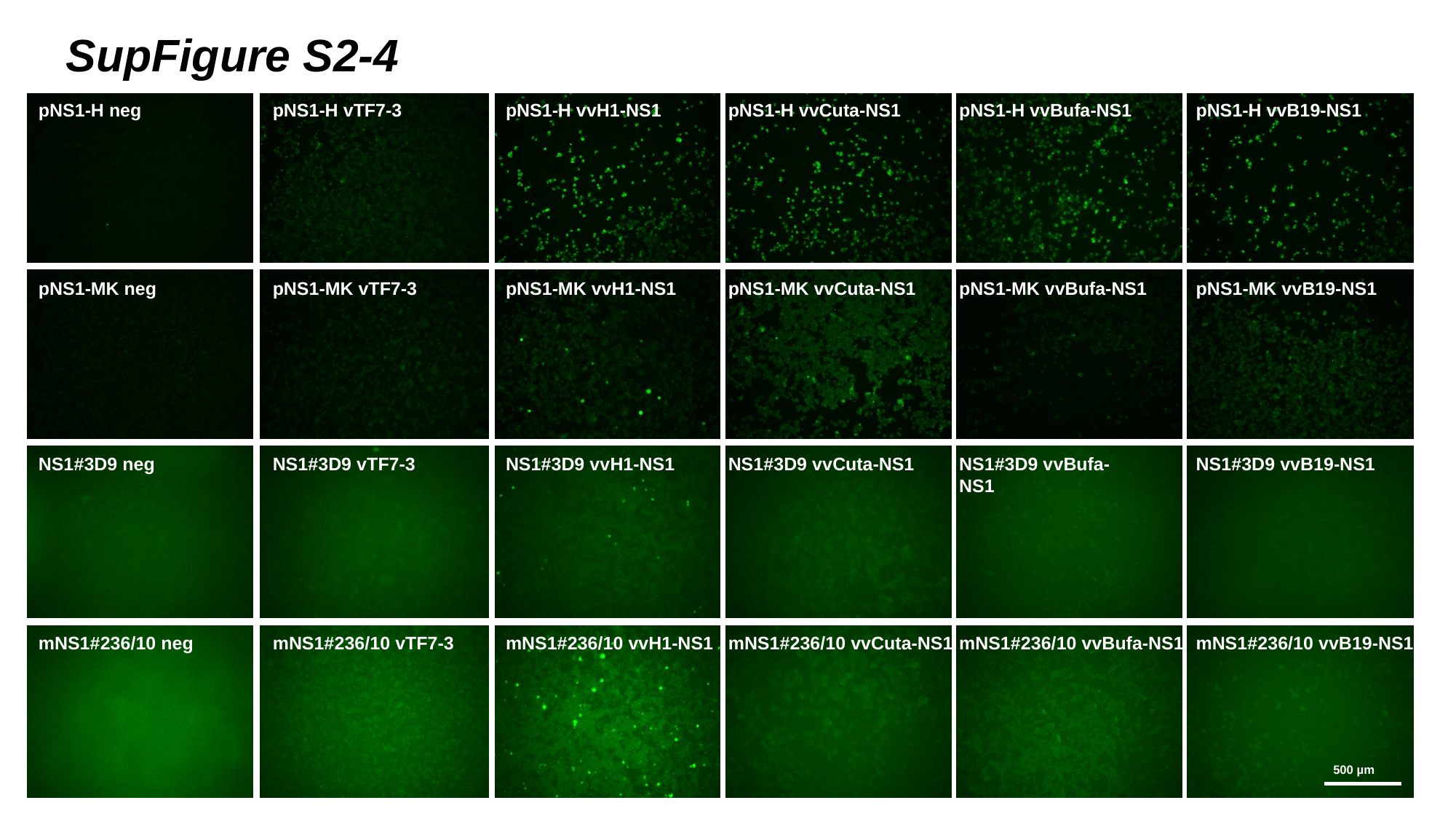

SupFigure S2-4
pNS1-H neg
pNS1-H vTF7-3
pNS1-H vvH1-NS1
pNS1-H vvCuta-NS1
pNS1-H vvBufa-NS1
pNS1-H vvB19-NS1
pNS1-MK neg
pNS1-MK vTF7-3
pNS1-MK vvH1-NS1
pNS1-MK vvCuta-NS1
pNS1-MK vvBufa-NS1
pNS1-MK vvB19-NS1
NS1#3D9 neg
NS1#3D9 vTF7-3
NS1#3D9 vvH1-NS1
NS1#3D9 vvCuta-NS1
NS1#3D9 vvBufa-NS1
NS1#3D9 vvB19-NS1
mNS1#236/10 neg
mNS1#236/10 vTF7-3
mNS1#236/10 vvH1-NS1
mNS1#236/10 vvCuta-NS1
mNS1#236/10 vvBufa-NS1
mNS1#236/10 vvB19-NS1
500 µm
