# Supplementary material for: Generation and Validation of Monoclonal Antibodies Suitable for Detecting and Monitoring Parvovirus Infections
Source: Pathogens. 2022 Feb 4;11(2):208. doi: 10.3390/pathogens11020208 (PMC8877868; doi:10.3390/pathogens11020208)
Supplement: Supplementary file 1 [file pathogens-11-00208-s001.zip › mAB-NS1 Sup Figure S3.pptx]

## Slide 1
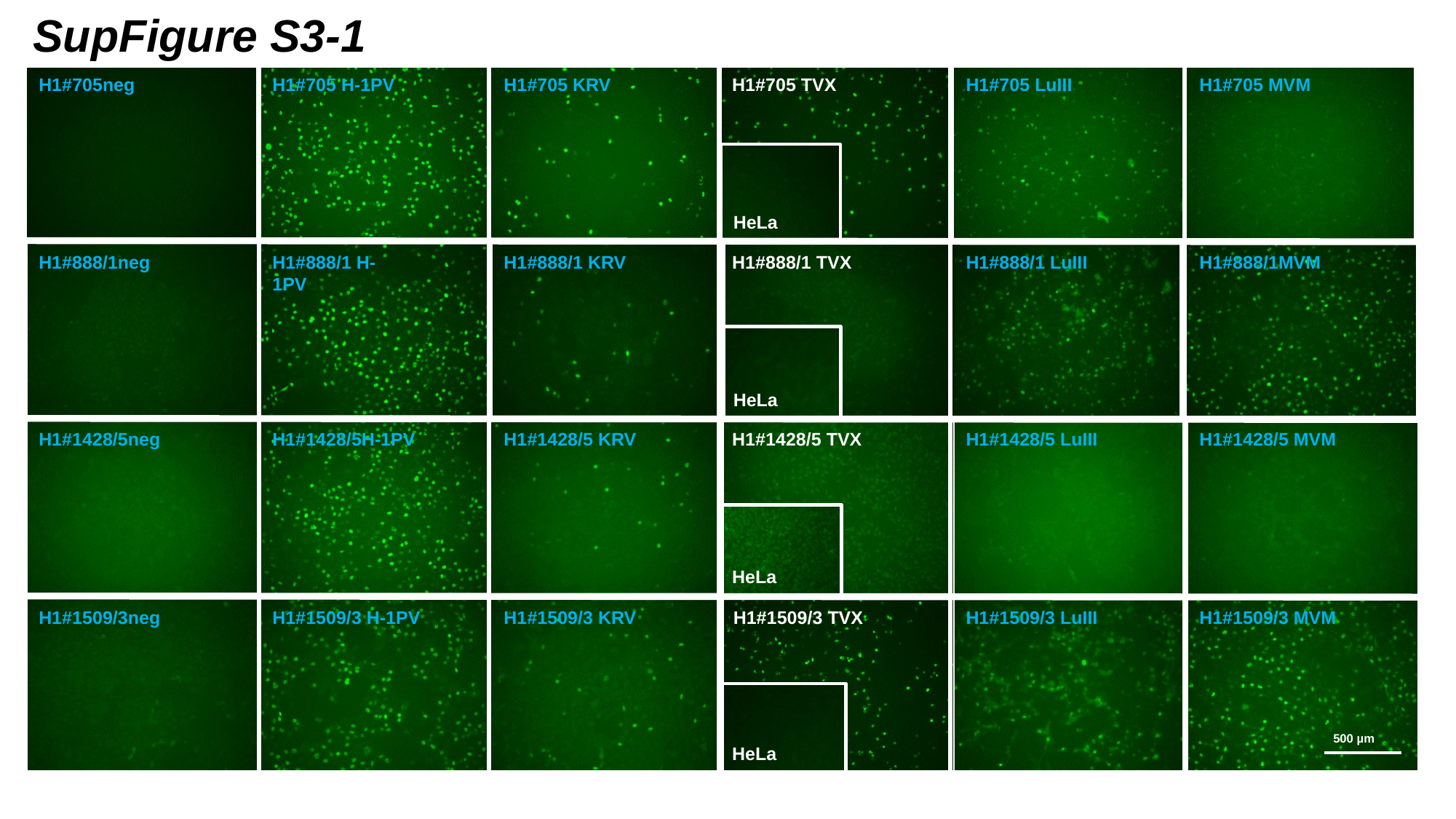

SupFigure S3-1
H1#705neg
H1#705 H-1PV
H1#705 KRV
H1#705 TVX
H1#705 LuIII
H1#705 MVM
HeLa
H1#888/1neg
H1#888/1 H-1PV
H1#888/1 KRV
H1#888/1 TVX
H1#888/1 LuIII
H1#888/1MVM
HeLa
H1#1428/5neg
H1#1428/5H-1PV
H1#1428/5 KRV
H1#1428/5 TVX
H1#1428/5 LuIII
H1#1428/5 MVM
HeLa
H1#1509/3neg
H1#1509/3 H-1PV
H1#1509/3 KRV
H1#1509/3 TVX
H1#1509/3 LuIII
H1#1509/3 MVM
HeLa
500 µm

## Slide 2
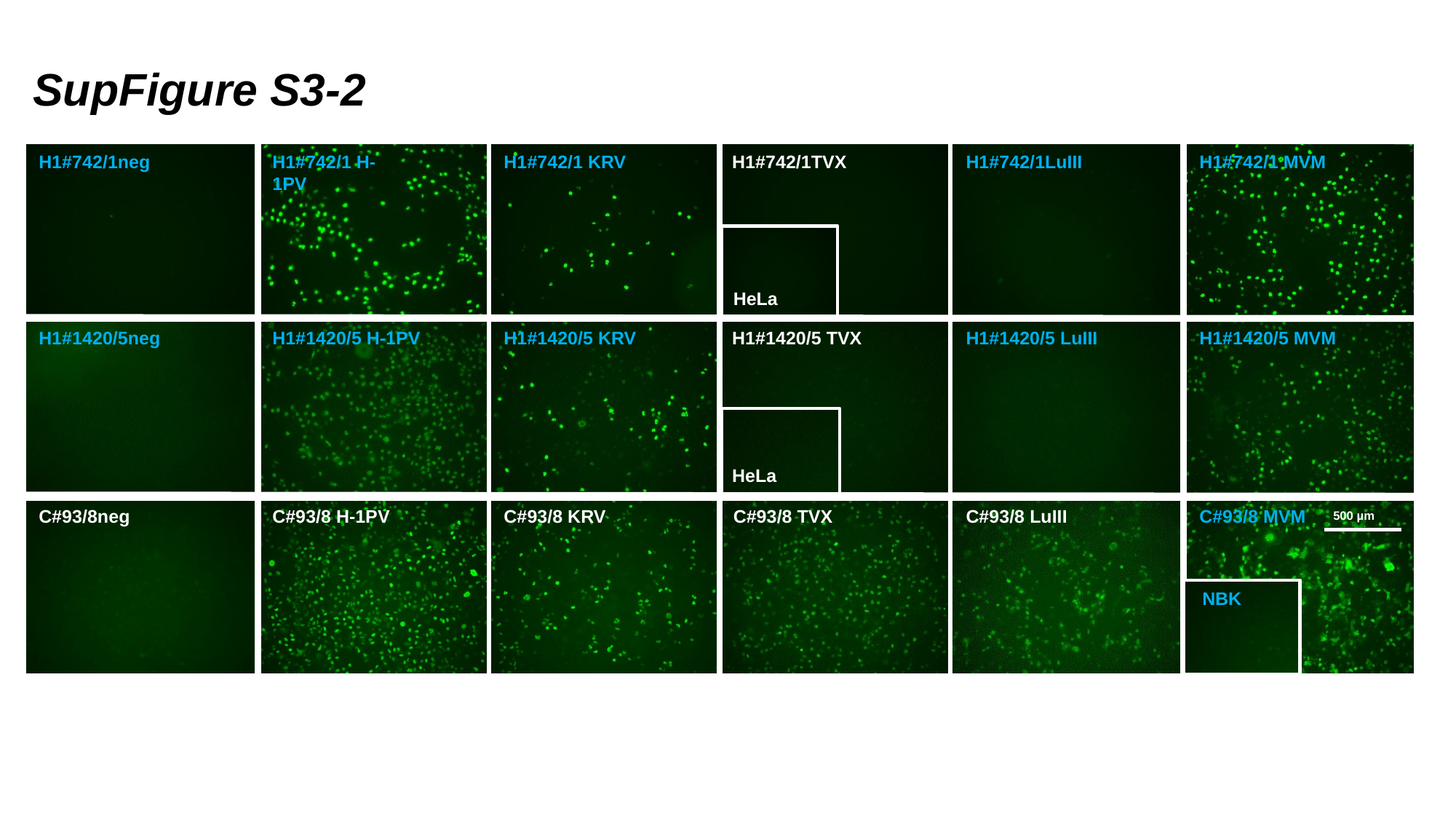

H1#742/1neg
H1#742/1 H-1PV
H1#742/1 KRV
H1#742/1TVX
H1#742/1LuIII
H1#742/1 MVM
HeLa
H1#1420/5neg
H1#1420/5 H-1PV
H1#1420/5 KRV
H1#1420/5 TVX
H1#1420/5 LuIII
H1#1420/5 MVM
HeLa
C#93/8neg
C#93/8 H-1PV
C#93/8 KRV
C#93/8 TVX
C#93/8 LuIII
C#93/8 MVM
NBK
SupFigure S3-2
500 µm
NBK

## Slide 3
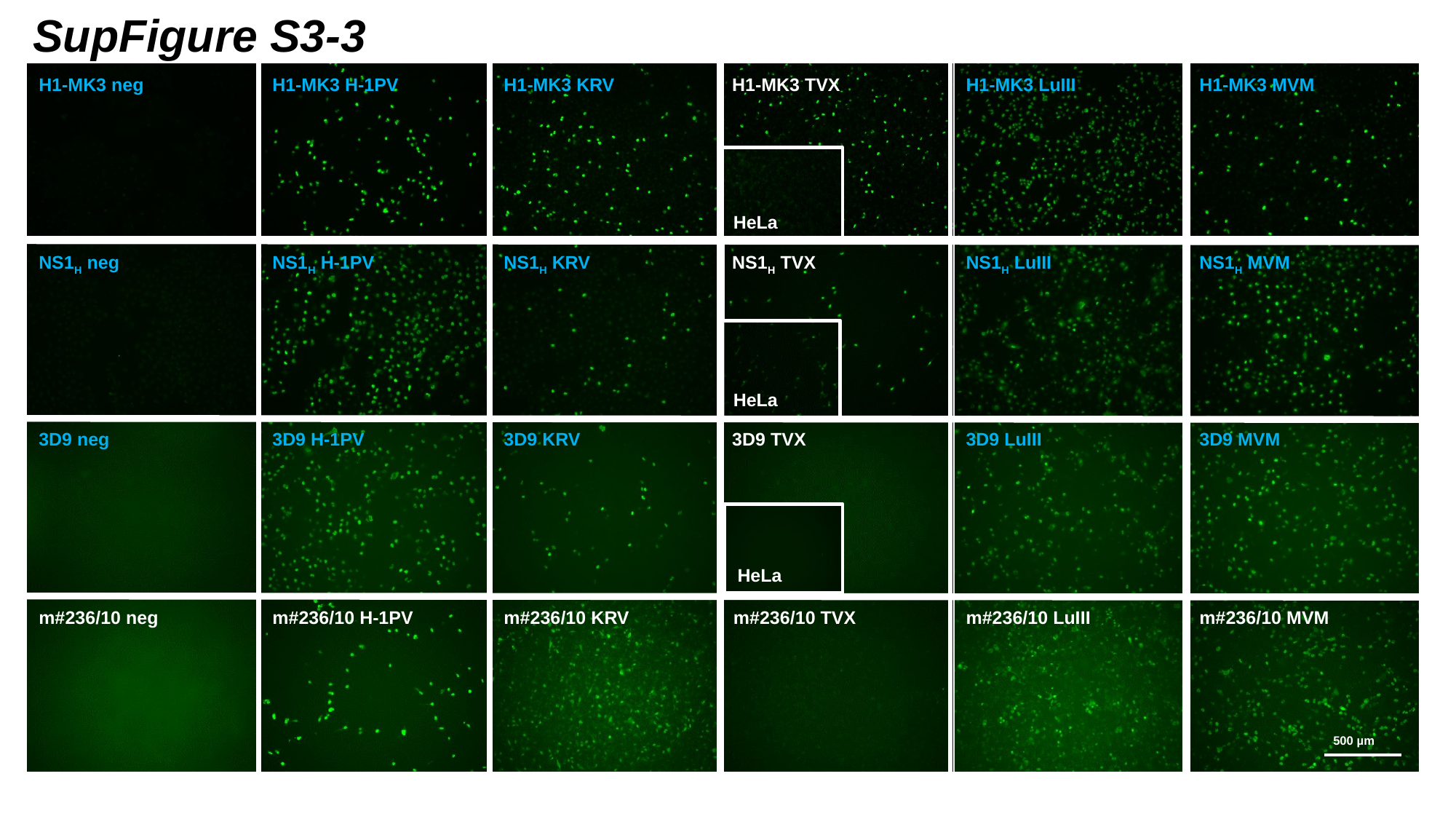

SupFigure S3-3
H1-MK3 neg
H1-MK3 H-1PV
H1-MK3 KRV
H1-MK3 TVX
H1-MK3 LuIII
H1-MK3 MVM
HeLa
NS1H neg
NS1H H-1PV
NS1H KRV
NS1H TVX
NS1H LuIII
NS1H MVM
HeLa
3D9 neg
3D9 H-1PV
3D9 KRV
3D9 TVX
3D9 LuIII
3D9 MVM
m#236/10 neg
m#236/10 H-1PV
m#236/10 KRV
m#236/10 TVX
m#236/10 LuIII
m#236/10 MVM
HeLa
500 µm
