# Supplementary material for: Generation and Validation of Monoclonal Antibodies Suitable for Detecting and Monitoring Parvovirus Infections
Source: Pathogens. 2022 Feb 4;11(2):208. doi: 10.3390/pathogens11020208 (PMC8877868; doi:10.3390/pathogens11020208)
Supplement: Supplementary file 1 [file pathogens-11-00208-s001.zip › mAB-NS1 Sup Figure S4.pptx]

## Slide 1
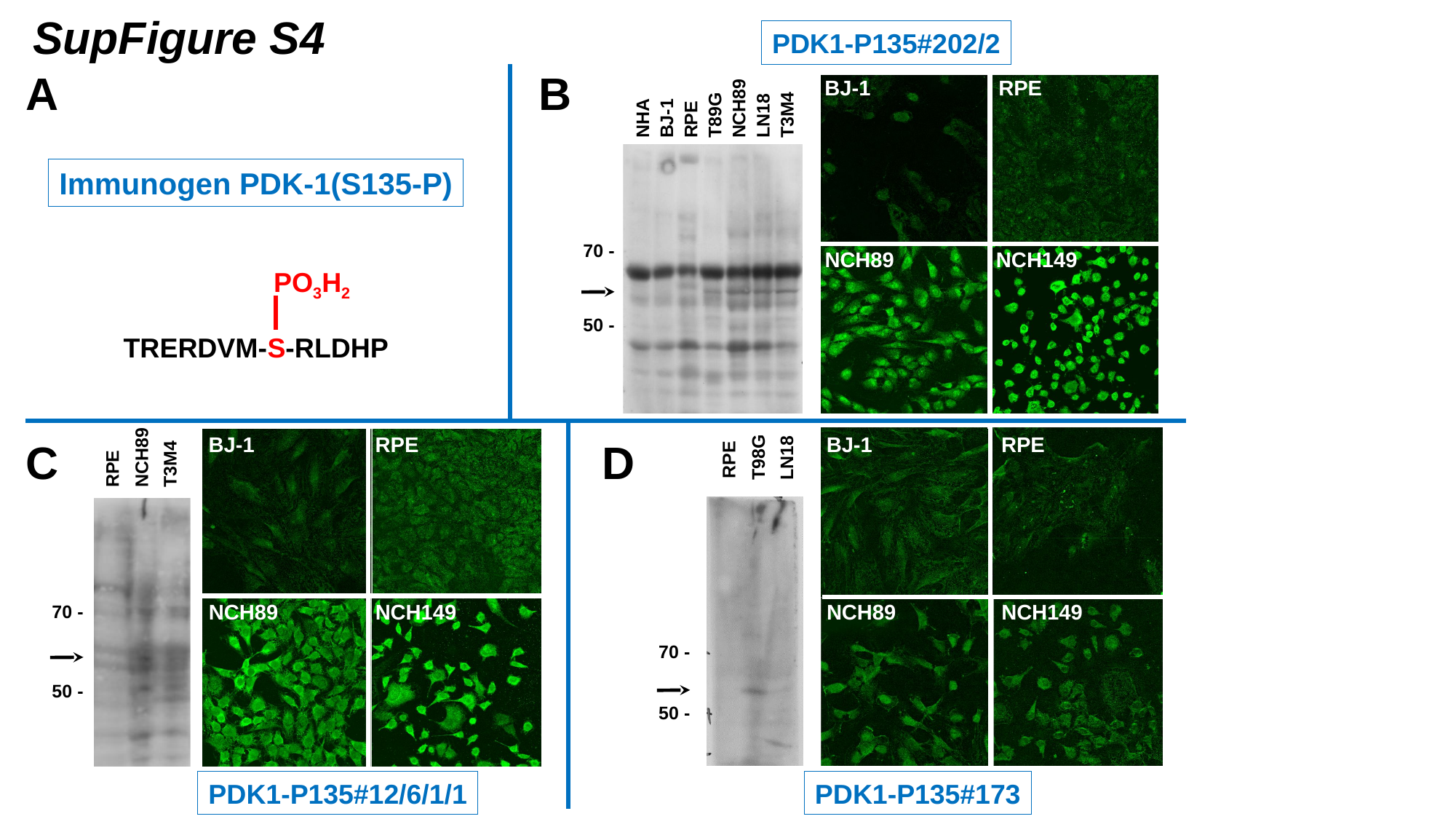

SupFigure S4
PDK1-P135#202/2
A
B
BJ-1
RPE
NCH89
T3M4
T89G
LN18
NHA
BJ-1
RPE
70 -
NCH89
NCH149
50 -
Immunogen PDK-1(S135-P)
PO3H2
TRERDVM-S-RLDHP
BJ-1
RPE
T98G
LN18
RPE
NCH89
NCH149
70 -
50 -
BJ-1
RPE
NCH89
T3M4
RPE
NCH89
NCH149
70 -
50 -
C
D
PDK1-P135#12/6/1/1
PDK1-P135#173
